# *In-silico* molecular modelling, MM/GBSA binding free energy and molecular dynamics simulation study of novel pyrido fused imidazo[4,5-*c*]quinolines as potential anti-tumor agents

**DOI:** 10.3389/fchem.2022.991369

**Published:** 2022-09-30

**Authors:** Upala Dasmahapatra, Chitluri Kiran Kumar, Soumyadip Das, Prathima Thimma Subramanian, Poornimaa Murali, Arnold Emerson Isaac, Karuppasamy Ramanathan, Balamurali MM, Kaushik Chanda

**Affiliations:** ^1^ Department of Chemistry, School of Advanced Sciences, Vellore Institute of Technology, Vellore, Tamil Nadu, India; ^2^ Department of Biotechnology, School of Bio Sciences and Technology, Vellore Institute of Technology, Vellore, Tamil Nadu, India; ^3^ Division of Chemistry, School of Advanced Sciences, Vellore Institute of Technology, Chennai campus, Chennai, Tamil Nadu, India

**Keywords:** PI3K, computational chemistry, cancer, heterocyclic, molecular dynamic (MD)

## Abstract

With an alarming increase in the number of cancer patients and a variety of tumors, it is high time for intensive investigation on more efficient and potent anti-tumor agents. Though numerous agents have enriched the literature, still there exist challenges, with the availability of different targets and possible cross-reactivity. Herein we have chosen the phosphoinositide 3-kinase (PI3K) as the target of interest and investigated the potential of pyrido fused imidazo[4,5-*c*]quinoline derivatives to bind strongly to the active site, thereby inhibiting the progression of various types of tumors. The AutoDock, Glide and the Prime-MM/GBSA analysis are used to execute the molecular docking investigation and validation for the designed compounds. The anti-tumor property evaluations were carried out by using PASS algorithm. Based on the GLIDE score, the binding affinity of the designed molecules towards the target PI3K was evaluated. The energetics associated with static interactions revealed **1j** as the most potential candidate and the dynamic investigations including RMSD, RMSF, Rg, SASA and hydrogen bonding also supported the same through relative stabilization induced through ligand interactions. Subsequently, the binding free energy of the Wortmannin and **1j** complex calculated using MM-PBSA analysis. Further evaluations with PASS prediction algorithm also supported the above results. The studies reveal that there is evidence for considering appropriate pyrido fused imidazo[4,5-*c*]quinoline compounds as potential anti-tumor agents.

## Graphical Abstract

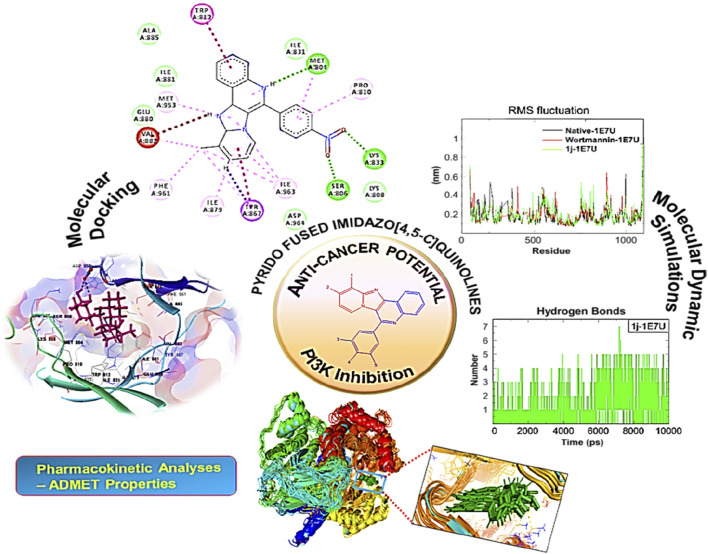

## Introduction

**FIGURE 1:**
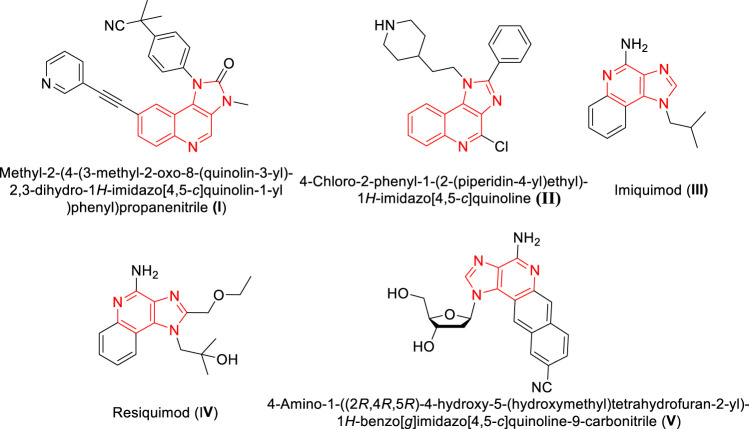
Structures of imidazo[4,5-c]quinolone moieties with phamacological and photophysical significance.

Many heterocyclic molecules particularly fused heterocyclic compounds are privileged structures that are being utilized extensively in various drug discovery programs. These molecules exhibit a broad spectrum of pharmacological properties with variations arising from the nature and position of the substituent in the heterocyclic scaffolds. Moreover, fused heterocyclic molecules with multiple rings formed *via* sharing of two atoms and a bond between the rings, are recognized as promising fluorescent probes materials. Fused heterocycles like indole, quinoline and isoquinoline possess unique properties with variations in their structural motifs and modified electronic environment. Compounds possessing such fused heterocyclic units exhibit a wide spectrum of biological and physical properties (Grzybowski et al., 2014; Sharma et al., 2021; Salehian et al., 2021). Recently, luminescent properties along with the possible applications of amino substituted benzimidazo[1,2-*a*]quinoline were reported through experimental and computational experiments (Hranjec et al., 2017). Pyrido fused imidazo[4,5-*c*]quinolones are a class of such fused heterocyclic structures that originated from the fusion of imidazo[1,2-*a*] pyridines with quinoline frameworks. Imidazo[4,5-*c*]quinoline is considered as a privileged scaffold with a wide range of pharmacological activity such as PI3K/PKB-pathway inhibitor (Stauffer et al., 2008) **(I)** TNF-α inhibitor (Izumi et al., 2003) **(II)**. Further, there are many immunomodulator drugs such as Imiquimod **(III)** and Resiquimod **(IV)** contains imidazo[4,5-c]quinolone moiety (Figure 1) that are available in the market (Dockrell and Kinghorn, 2001). Recently Siraiva *et al* have developed a novel fluorescent benzoimidazo[4,5-c]quinoline nucleoside, exhibiting distinct pH dependent photophysical properties (Siraiwa et al., 2016). Recently, Szostak *et al* reported the importance and synthetic route to quinolone moiety as fused heterocyclic compounds (Zhang et al., 2020). These molecules are reported to serve as bio-probes for nucleic acid structure and genetic analyses (Jones et al., 2015).

Though numerous small-molecular fluorescent probes are available as promising probes in chemical biology, the search for simple fluorogenic scaffolds with the possibility of rational design and predictable photophysical properties for high-throughput screening is still under intense investigation.

All these were proven to be possible with structurally diverse quinoline-based fluorophores and were successfully implemented for live cell imaging applications. (a)

With an alarming increase in the number of cancer patients and variety of tumors, it is high time for intensive investigation on more efficient and potent anti-cancer agents. Though numerous agents have enriched the literature, still there exist challenges, with the availability of different targets and cross-reactivity. Herein we have chosen the phosphoinositide 3-kinase (PI3K) as the target of interest and investigated the potential of pyrido fused imidazo[4,5-*c*]quinoline derivatives to bind strongly to the active site, thereby inhibiting the progression of various types of tumors. For instance, Phosphoinositide 3-kinases (PI3Ks) are broadly classified into three classes namely, Class I (A and B), II (A and B) and III, amongst these class I PI3Ks were extensively studied for their dysregulation in several cancer types. (b) Specifically, the isoform of PI3K-γ has extended its role in uncontrolled growth and metastases in various cancer types including breast cancer and hematological malignancies (Schmid et al., 2013). Literature evidence has identified the role of PI3K-γ as a molecular switch between immune stimulants and suppressors during inflammation and cancer (Kaneda et al., 2016). Thus, in the present study, a specific isoform of PI3K-γ was targeted to identify potential and selective inhibitors. Furthermore, limiting PI3K-γ expression to the hematopoietic system may reduce the toxicity of specific inhibitors compared to pan-PI3K inhibitors. There are many literature reports indicating the potential of imidazo[4,5-*c*]quinoline derivatives towards protein kinase inhibition particularly those involved in malignant inducing pathological processes (Alemi et al., 2022; Zaryouh et al., 2022). It is known that alterations in the enzymatic activity of PI3K induce multiple diseases, ranging from cancer to chronic inflammation (Brauer et al., 2012; Shymanets et al., 2015). PI3K signalling plays key roles in cellular responses including proliferation, protein synthesis and vesicular trafficking. It is also reported that imidazo[4,5-*c*]quinolines reduce the cellular toxicity in host systems and paves way for its selectivity over human kinases (Nguyen et al., 2021; Wright et al., 2021). Additionally, we think that the fusion of two significant bioactive compounds, such as imidazo[1,2-a]pyridines and quinolines, results in an intriguing bioactive property. Recently, we have reported the anti-tumor activities of substituted 2*H*-indazole derivatives (Panchangam et al., 2019) and novel C,N-cyclometalated 2*H*-indazole-ruthenium(II) and -iridium(III) complexes targeting triple negative breast cancer cells (Rao et al., 2020; Panchangam et al., 2021).

In continuation of our ongoing drug discovery programme for finding new leads for different diseases through computational approach, (c) we have investigated the potential of pyrido fused imidazo[4,5-*c*]quinoline derivatives as inhibitor for tumor progression through various *in silico* approaches including docking, molecular dynamic and pharmacokinetic evaluations.

## Methods and methods

### Compilation of dataset

**FIGURE 2:**
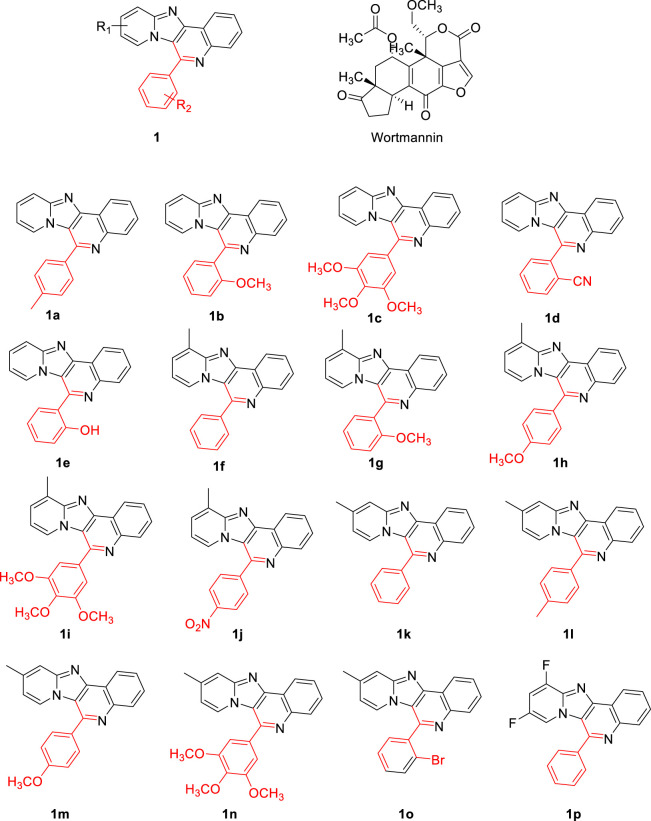
The chemical structures of the evaluation of proposed pyrido fused imidazo[4,5-*c*]quinoline derivatives.

The 3 dimensional structure of PI3K protein in complex with wortmannin (PDB ID: 1E7U) was obtained from Brookhaven Protein Data Bank and used for docking studies after certain refinements. All the water molecules and other bound ligands were deleted from the PDB structure followed by the addition of hydrogen atoms to the protein molecule. AutoDockTools-1.5.6 was utilized to assign kollman charges (Chandra Singh and Kollman, 1984) to all atoms of the protein. Conventional procedures were employed to refine protein structures. ChemDraw Ultra 8.0 tool was used to draw the structures of pyrido fused imidazo[4,5-*c*]quinoline derivatives and the reference (Wortmannin) employed in future processing as shown in Figure 2. Wortmannin is a steroid metabolite present in fungus and is well investigated as a potential inhibitor for PI3K. The PDB 1E7U structure taken from the database is available as bound with Wortmannin. Moreover, the substitutions were chosen based on the electron donating or withdrawing potential at ortho- and para positions of the aromatic ring so as to observe push-pull mechanism along the single rotatable bond that connects the pyrido fused imidazo[4,5-*c*]quinoline. The geometry of ligands was optimized following DFT calculations with B3LYP functional and 6-311++g** basis set followed by the assignment of Gasteiger partial atomic charges (Frisch et al., 2010). AutoTors was used to define possible flexibility and torsions. The binding pocket was identified using online active site identifier tool and a grid box was constructed. *AutoGrid* program pre-calculates *Grid maps*. Using Lamarckian genetic algorithm along with AMBER force field-based energy assessments, various possible conformational states of ligands were explored. Docking calculations were performed with default parameters. The binding energy was evaluated using the following scoring function.

$$∆G={∆G}_{vdw}+{∆G}_{hbond}+{∆G}_{elec}+{∆G}_{tor}+{∆G}_{desolv}$$

The free energy changes associated with the binding of flexible ligand to rigid target proteins were calculated with the above equation that includes contributing parameters like *van der* Waals dispersion/repulsion (ΔG_vdw_), electrostatic (ΔG_elec_) and hydrogen bonding interaction (ΔG_hbond_), torsional constraints (ΔG_tor_) and desolvation effects (ΔG_sol_).

### Drug-likeliness, molecular docking and binding energy calculations

The designed compound’s pharmacodynamic and pharmacokinetic properties have been assessed through using QikProp module (Wang et al., 2015). Lipinski rule of five (Ro5), Central Nervous System activity (CNS), and Human Oral Absorption (HOA) properties have been used to identify drug-like lead compounds (Hodgson, 2001). Moreover, the drug design tool MolAICal was employed to filter out the Pan-Assay Interference Compounds (PAINS) from the proposed candidates (Bai et al., 2021). The designed compounds have been attributed to docking analysis. It is an appropriate approach for investigating the mechanisms of interaction between the ligand and protein of interest. Molecular docking analysis was carried out using Autodock tools 1.5.6 and Glide algorithm of Schrodinger to calculate and retrieve the binding pose of the ligand molecules investigated in our study. In particular, the glide algorithm is of very much useful to discriminate the binders from non-binders. (d)

The free energy of the binding (ΔG bind) for the ligand molecules were then calculated by employing Prime MM-GBSA algorithm using the docked pose retrieved from Glide algorithm. (c) The binding pose of the complex structures were visually inspected by utilizing ligand interaction diagram tool of Schrodinger to gain insight into the binding mode.

The formula corresponds to the (ΔG bind) is given below:

ΔG(bind)=ΔG(solv)+ΔE(MM)+ΔG(SA)

Where, ΔG(solv) is the difference in GBSA solvation energy of the protein-ligand complex and the sum of the solvation energies for unliganded protein and ligand. ΔE(MM) is a difference in the minimized energies between protein-ligand complex and the sum of the energies of the unligated protein and ligand. ΔG(SA) is a difference in surface area energies of the complex and the sum of the surface area energies for the unligated protein and ligand. It also calculates the ligand strain energy by placing ligand in a solution which was auto-generated by VSGB 2.0 solvation model.

The molecular docking is performed on the protein with PDB ID 1E7U. The autogrid was generated centring 23.65, 61.82 and 19.28 (x, y, z coordinate) with grid size of 60, 52, 52 (x, y, z). The docking calculations were carried out by general algorithm parameters with 10 runs of population size 150. The maximum number of evaluations was set to be 25,000 000 (long) with the rate of gene mutation 0.02 and the rate of crossover mode 0.8.

### Molecular dynamic simulations

The GROMACS 2018 version was used to analyse the dynamic characteristics of molecules from within the biomolecular system. First, the Prodrg server was used to implement the GROMOS force field and prepare the ligand topology (Sargsyan et al., 2017). The SPC (single point charge) water model included within the dodecahedral box was used to solvated both complexes. Additionally, the steepest decent algorithm was used to carry out energy minimization for each complex. Using the particle mesh ewald (PME) method and the LINCS algorithm respectively, the electrostatic and bond length computations were enforced (Schüttelkopf and Van Aalten, 2004; Wang et al., 2010). The SHAKE algorithm was used to measure the restricted hydrogen bond length (Amiri et al., 2007). Each compound was equilibrated for 100 ps using the NVT and NPT isothermal-isobaric ensemble. Subsequently, a 100 ns MD simulation with a 2 fs integrative step was performed. By utilising the GROMACS toolbox to calculate the development of RMSD and hydrogen bonds, the conformational stability of the protein-ligand complex was investigated in the current study.

### Molecular mechanics Poisson–Boltzmann surface area (MM-PBSA) studies

MM-PBSA calculations helps in integrating the high-throughput molecular dynamics (MD) simulations with binding free energies of the protein-ligand interaction, that includes interaction free energies such as van der waals, electrostatic, polar salvation, SASA and binding energies. In this study we have adopted g_mmpbsa package developed by Kumari *et al.* (Kumari et al., 2014) We executed g_mmpbsa in single step calculation method by using the protein-ligand MD trajectory files along with parameter file. Using MmPbsaStat.py python script provided by g_mmpbsa package van der waals, electrostatic, polar salvation, SASA and binding energies are determined. In addition, we have predicted the contribution energy of each residue by executing MmPbSaDecomp.py of g_mmpbsa package.

## Results and discussion

### Molecular docking and MM-GBSA calculations

The Glide module of Schrödinger software was employed to estimate the binding affinities of each ligand molecule towards the PI3K target. The binding poses of each small molecule were ranked and scored using XP GScore. The results of molecular docking for 16 compounds were tabulated in Table 1. The binding energy calculations were performed with Molecular Mechanics-Generalized Born Surface Area (MM-GBSA) protocol using VSGB solvation model. MM-GBSA rescoring analysis was carried out to eliminate false positive predictions. Wortmannin was used as control while performing docking calculations. The XP scores of the small molecules varied from −5.68 to −2.56 kcal mol^−1^. The free binding energy of all the complexes and individual contributions for the total energy values are given in Table 1. From Table 1, it is clear that compound 1j was found to have better XP GScore than other molecules when compared with reference compound (wortmannin: −4.37 kcal mol^−1^).

**TABLE 1:** The free energy (∆G) parameters and the binding score of various pyrido fused imidazo[4,5-*c*]quinoline derivatives with PI3K as obtained from Glide docking module. All the energies are reported in kcal mol^−1^.

Compounds	Docking score	ΔG_bind_	ΔG_bind_ Covalent	ΔG_bind_Lipo	ΔG_bind_Solv GB	ΔG_bind_vdW	Ligand strain energy
*Wortmannin*	*−4.37*	*−62.03*	*4.95*	*−40.74*	*22.79*	*−44.1*	*4.94*
1a	−3.27	−68.79	6.42	−65.71	25.55	−36.04	11.31
1b	−5.09	−59.92	2.96	−53.72	27.58	−34.63	3.86
1c	−4.46	−77.24	4.76	−69.47	31.25	−47.59	7.55
1d	−4.77	−63.27	2.80	−49.31	32.13	−36.72	4.52
1e	−4.60	−54.82	1.60	−51.48	29.27	−36.35	5.51
1f	−4.73	−74.16	0.92	−61.09	24.87	−39.27	2.12
1g	−4.36	−55.25	12.19	−59.59	29.22	−35.68	18.08
1h	−4.40	−77.49	3.31	−66.58	30.45	−39.43	4.97
1i	−4.91	−67.33	6.65	−61.64	34.29	−35.99	10.85
**1j**	−**5.68**	−**57.80**	**8.60**	−**55.10**	**29.50**	−**43.22**	**14.37**
1k	−4.47	−58.44	2.84	−55.35	33.13	−36.26	4.95
1l	−4.34	−67.34	0.08	−58.74	33.67	−42.35	0.71
1m	−2.56	−65.99	3.55	−57.87	35.35	−41.98	6.76
1n	−3.44	−57.60	−0.79	−52.44	34.82	−31.07	−0.39
1o	−4.42	−74.15	2.15	−59.28	31.74	−44.47	3.93
1p	−5.22	−67.09	2.14	−58.50	33.07	−34.14	5.11

**FIGURE 3:**
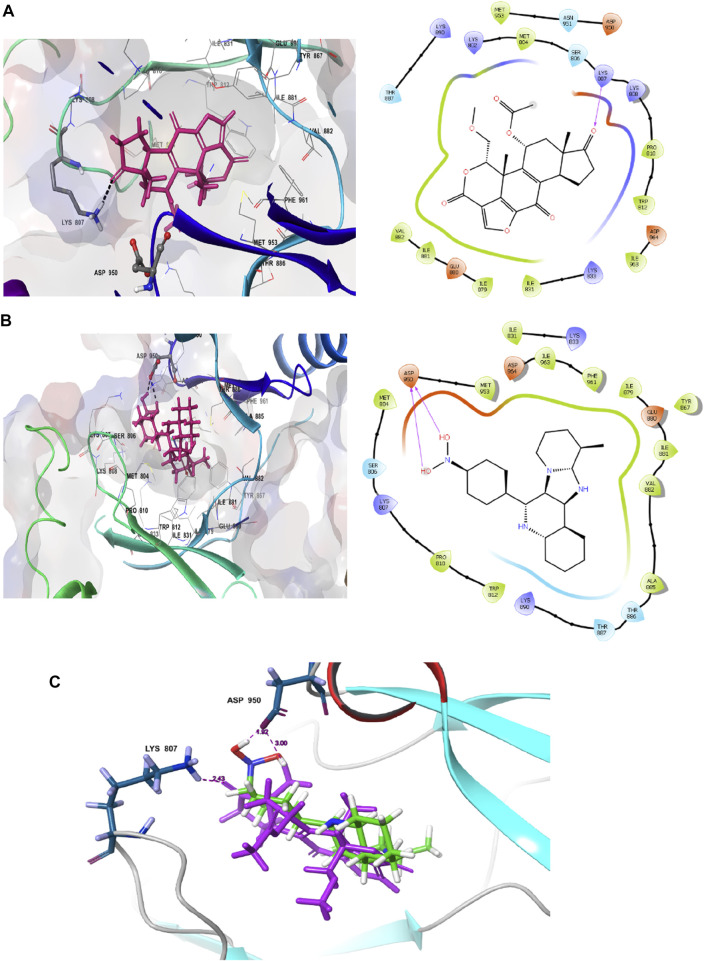
Ligand interaction analysis of the **(A)** Wortmannin, **(B)** compound **1j** and **(C)** The superimposed view of wortmannin (green) and **1j** (violet) with target receptor.

The interaction pattern of the compound **1j** were analysed to gain insights into the binding characteristics of small molecules. The binding pattern of the reference and the compound **1j** were highlighted in Figure 3. From Figure 3 it is evident that the reference compound Wortmannin was capable of forming hydrogen bond interaction with the LYS 807 residue. On the other hand, the hit molecule **1j** was found to interact with the target protein *via* two hydrogen bond interactions with residue ASP 950. The increased hydrogen bond formation by the hit molecule indicates its enhanced binding affinity towards the target protein.

**FIGURE 4:**
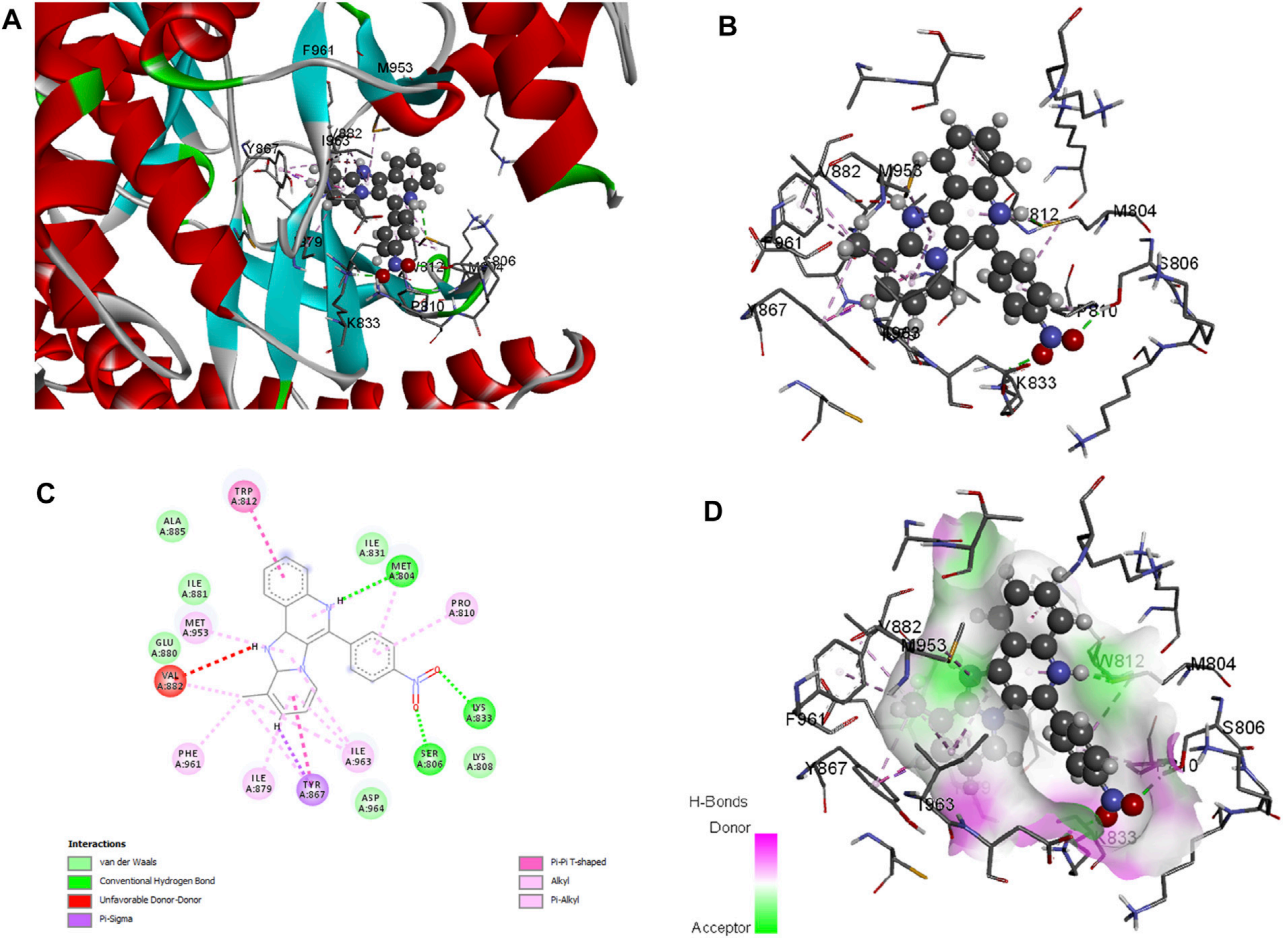
Docked conformation of compound 1j with crystal structure of phosphoinositide 3-kinase. (Pdb id: 1E7U) is shown. 1j is represented as ball and stick. **(A)** 3D Ribbon representation of the binding cavity and its interacting residues with 1j. **(B)** 3D stick representation of the binding cavity and its interacting residues with 1j. **(C)** 2D representation of the binding cavity residues along with the nature of interactions. **(D)** Binding cavity highlighting hydrogen bonding interactions with the residues with 1j.

Molecular docking is employed in the advanced drug development process to accurately calculate the binding efficacy of derivatives. Additionally, the Autodock tools 1.5.6 algorithms were employed to calculate the total binding affinity of **16** molecules to the active site of the target P13K. The binding affinity and other features offered by AutoDock algorithm are summarised in Table 2. The binding affinities of all 16 ligands ranged from −6.32 to −7.65 kcal mol^−1^. In particular, **1j** shows good binding affinity to the target site compared to other compounds. Docked conformation of other pyrido fused imidazo[4,5-c]quinolines inhibitors with PI3K along with geometry optimized coordinates are shown in Supplementary Figure S1 (supporting information). The interaction between the hit compound and the aminoacid residues in the active site was also identified and demonstrated using the BIOVIA discovery studio visualizer as shown in Figure 4.

**TABLE 2:** The binding energy (∆G_BE_) and intermolecular energy (∆G_intermol_) ofpyrido fused imidazo[4,5-*c*]quinoline derivativeswith PI3K as obtained from AutoDock Tools are given below. All the energies are reported in kcal mol^−1^.

Compounds	Binding energy	Intermolecular energy	Vdw_hb_desol_energy	Electrostatic energy	Torsional energy
*Wortmannin*	*−7.82*	−*8.72*	*−8.60*	*−0.12*	*0.89*
**1a**	−6.80	−7.09	−7.01	−0.08	0.30
**1b**	−6.57	−7.17	−7.15	−0.02	0.60
**1c**	−6.71	−7.90	−7.96	0.05	1.19
**1d**	−6.63	−7.23	−7.17	−0.05	0.60
**1e**	−6.34	−6.34	−6.31	−0.03	0.00
**1f**	−6.75	−7.05	−7.02	−0.03	0.30
**1g**	−6.63	−7.23	−7.21	−0.02	0.60
**1h**	−6.80	−7.39	−7.43	0.03	0.60
**1i**	−6.80	−7.99	−8.06	0.07	1.19
**1j**	−**7.65**	−**8.24**	−**6.32**	−**1.92**	**0.60**
**1k**	−6.59	−6.89	−6.85	−0.04	0.30
**1l**	−7.10	−7.40	−7.33	−0.07	0.30
**1m**	−7.10	−7.40	−7.33	−0.07	0.30
**1n**	−6.88	−8.08	−8.14	0.06	1.19
**0**	−7.16	−7.46	−7.42	−0.04	0.30
**1p**	−6.32	−6.62	−6.60	−0.02	0.30

Based on the above evaluations, compound **1j** was found to exhibit lowest free energy values upon interacting with PI3K protein, and the resulting complex was stabilized by van der Waals energy contribution though the ligand strain energy contribution was observed to be high. Thus, our pipelines comprised of multiple docking and MM-GBSA analysis will provide clear view of anti-cancer activity of compound **1j** through *in silico* study. Figures 3A,B represents the 2D docking of Wortmannin and **1j** with PI3K protein respectively using Glide module of Schrodinger suite. The interaction pattern also provides the insight into the binding pattern of the molecule. It depicts that compound **1j** is able to exhibit more intermolecular hydrogen bonding interaction than Wortmannin in the binding pocket of PI3K protein. Thus, we hypothesize that the increased interaction of ligand molecules with PI3K facilitates the effective binding of the designed molecule. Ligand interaction analysis of all compounds with target receptor are shown in Supplementary Figure S2 (supporting information).

### Molecular dynamic simulation

Detailed information on the drug-receptor interactions including binding affinity and orientation of the potent therapeutic molecules to the binding pocket were obtained from molecular docking investigations. Based on the binding affinity score, **1j** identified as the most potent inhibitor for PI3K protein towards tumor management. To investigate the binding interactions of any biological molecules in a biological process it is essential to understand the associated mechanisms which in turn can be achieved by molecular dynamic simulation (MDS) method. In the present study, the contacts made between the ligands **1j** and Wortmannin with protein PDB ID:1E7U were analyzed by static and dynamic studies. Molecular dynamics (MD) simulation was performed using GROMACS 2018 package for native protein 1E7U, 1j–1E7U complex, and Wortmannin-1E7U complex. To minimize the steric factors, energy minimization was carried out with the geometry of the complex 1j-1E7U through 50000 steps. The calculated average energy was found to be −498.24 × 10^3^ kcal mol^−1^ while that for the complex Wortmannin-1E7U was calculated to be −472.48 × 10^3^ kcal mol^−1^. This was followed by NVT and NPT to equilibrate the system. After a successful setup of the parameters, MD simulation was carried out for a 100 ns timescale period. In the present study we have determined Root mean square deviation (RMSD), Root mean square fluctuations (RMSF), Radius of gyration (Rg), Solvent accessible surface areas (SASA), and Hydrogen bonds (Hb) and further the results were analysed to dissect the actual mechanisms involved in the above interactions. These results are depicted by plotting graphs for respective analysis using Qtgrace.

### RMSD

**FIGURE 5:**
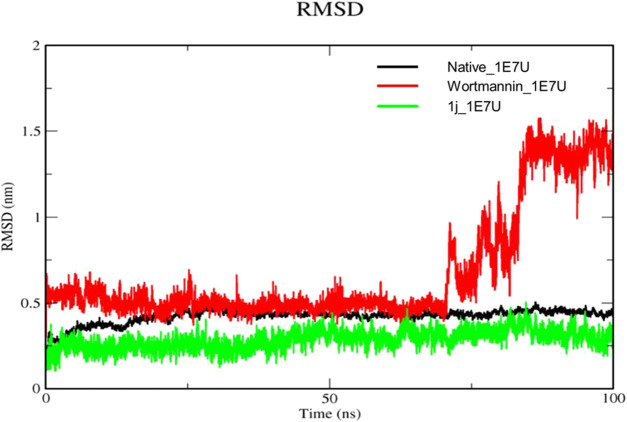
RMSD analysis for the native structure of 1E7U (black) and its complex with Wortmannin (red) and **1j** (green).

The Root mean square deviation (RMSD) is used to study the conformational stability of biological molecules (Zaki et al., 2021). The structural changes in 1E7U upon interacting with Wortmannin and **1j** ligands were determined by invoking RMSD analysis and the same is depicted in Figure 5. The overall stability and structural convergence of the two complexes Wortmannin-1E7U and **1j**-1E7U alongside native system were analyzed for ∼100 ns Initially, the Wortmannin–1E7U complex showed deviation till 0.60 nm and maintained its stability with minimal fluctuations till ∼68 ns and thereafter sudden instability was observed till ∼80 ns with maximum peak difference ∼1.12 nm (1.62 – 0.5 nm) and later became stable till 100 ns with minimal deviations. The overall average RMSD is calculated to be 0.6769 nm (Wortmannin–1E7U). Whereas complex **1j**–1E7U maintained stability throughout the simulation with a lower average of 0.2867 nm RMSD. Similar pattern of deviation was observed even in the case of native protein.

### RMSF

**FIGURE 6:**
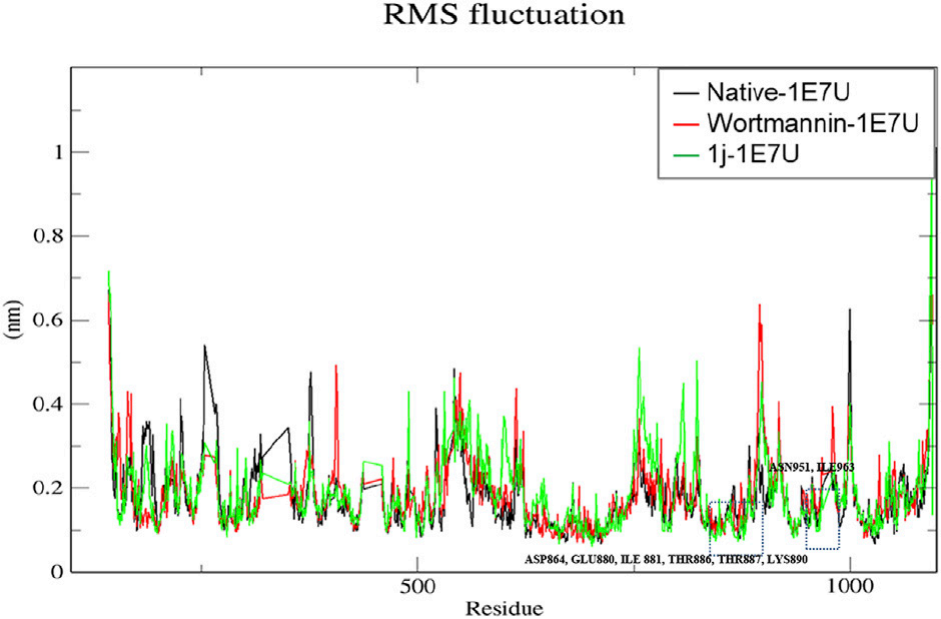
RMSF analysis for the native structure of 1E7U and its complex with wortmannin (red) and **1j** (green).

The root mean square fluctuation (RMSF) analysis represents the flexibility of each residue in the protein. Here we have examined the positional fluctuations of each protein residue in the native and its complexes with respective ligands (Baildya et al., 2021). Native protein 1E7U showed average fluctuations 0.173 nm whereas complex Wortmannin–1E7U was calculated to be 0.183 nm and complex 1j–1E7U evaluated as 0.185 nm. It can be observed from Figure 6 that residues in the binding site ASN951, ASP864, THR887, LYS890, THR886, TRP812, GLU880, ILE 881, ASN 951 and ILE963 display lower fluctuations.

### Radius of gyration (Rg)

**FIGURE 7:**
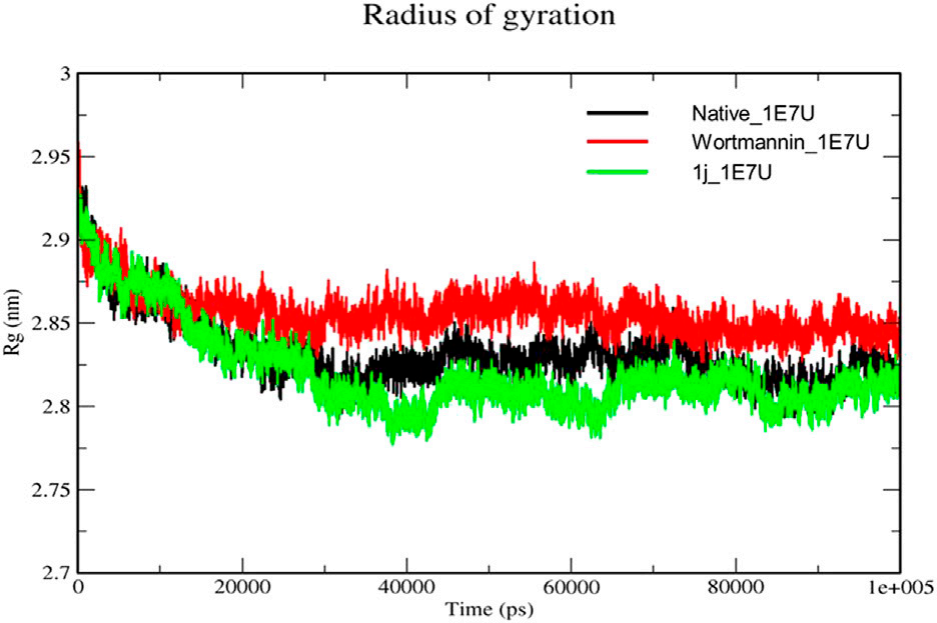
Radius of gyration analysis for the native structure of 1E7U (black) and its complexes with Wortmannin (red) and **1j** (green).

The Radius of gyration (Rg) is a measurement of the structural compactness of protein. The distance between the axis of the structure and the point of the atom during the rotation where maximum energy is transferred gives Rg (Khare et al., 2021). The ligand induced conformational changes alter the Rg upon binding to the target. In the present study, we have measured the Rg of native protein and the protein complexes Wortmannin–1E7U and 1j–1E7U to understand the compactness of the protein upon ligand interactions and the same is depicted in Figure 7. The Rg for native protein was calculated and the average Rg was found to be 2.8322 nm, whereas for the complexes Wortmannin–1E7U and 1j–1E7U were calculated to be 2.8563 nm, and 2.8208 nm respectively. There was a significant decrease in the Rg from 2.95 to 2.77 nm till ∼38 ns and maintained lower than native and Wortmannin complex with a difference of 0.0241 nm indicating that the compactness marginally increased upon interacting with **1j**.

### Solvent-accessible surface area (SASA)

**FIGURE 8:**
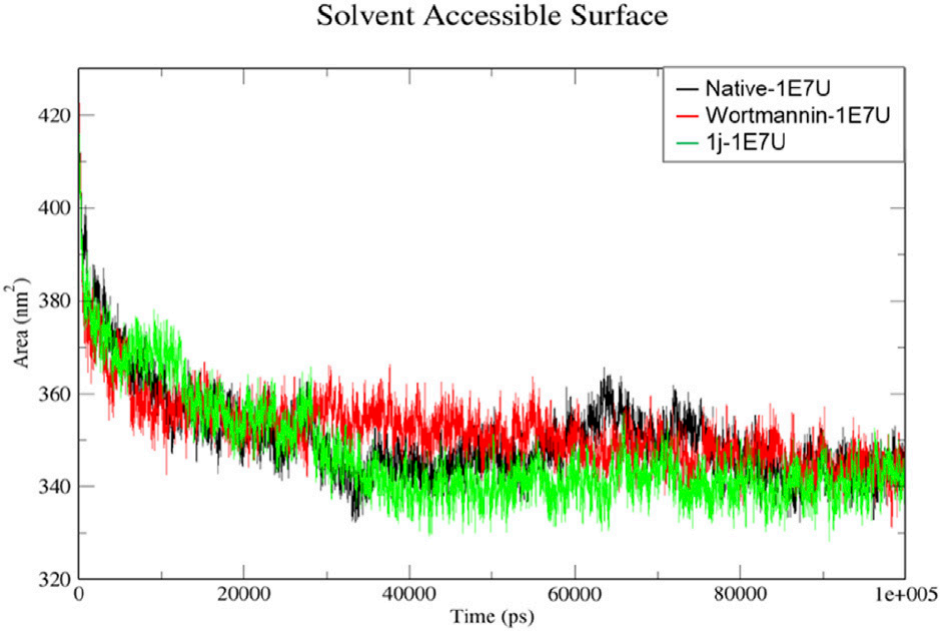
SASA analysis for the native structure of 1E7U (black) and its complex with Wortmannin (red) and **1j** (green).

The solvent-accessible surface area (SASA) is a method used to calculate polar and non-polar molecular surface area to understand the residues interacting with the surrounding solvent (Anuar et al., 2021). From Figure 8, the SASA for the native 1E7U, Wortmannin–1E7U complex, and 1j-1E7U complex where native protein average SASA was calculated to be 350.49, 352.038 and 347.148 nm^2^ respectively. It is observed that the 1j-1E7U complex possess lower SASA in comparison to the native and Wortmannin complex, thus signifying the conformational changes induced by **1j**.

### Hydrogen bond

**FIGURE 9:**
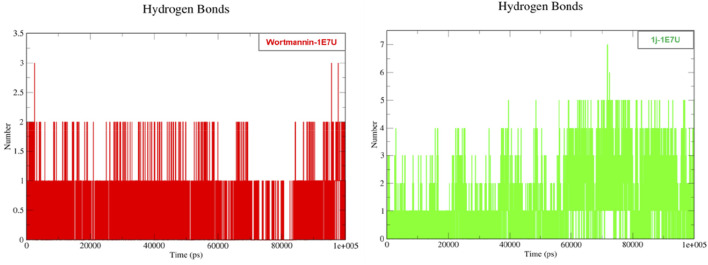
Hydrogen bond analysis throughout the 100 ns - 1E7U with Wortmannin (red), 1E7U with **1j** (green).

Hydrogen bonding is a geometrical analysis that is performed to understand the interactions of bio-molecules. Bio-molecules maintain their structural stability through various interactions and one of the crucial interactions is hydrogen bonding (Vora et al., 2020). In this study, we have examined the interactions of the native 1E7U with ligands Wortmannin and **1j** during 100 ns MD simulation and the same is depicted in Figure 9. The Wortmannin complex showed three hydrogen bonds out of which two are continuous throughout the simulation and the **1j** complex was observed to have seven hydrogen bonds out of which four were seen throughout the simulation and one hydrogen bond was formed after 60 ns and maintained contact till 100 ns The overall analysis depicts that **1j** is making more number of hydrogen contacts with 1E7U to maintain stability.

**FIGURE 10:**
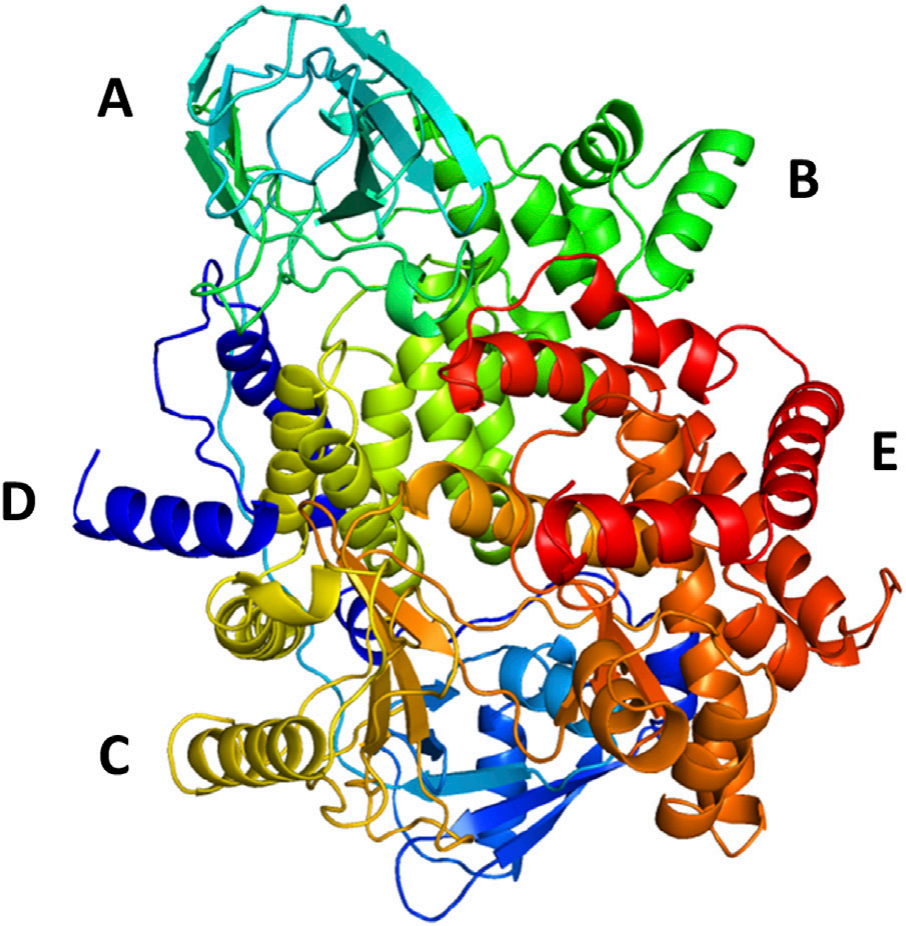
3D - Domain organization of the 1E7U **(A)**- phosphoinositide 3-kinase C2 (PI3K_C2) (skyblue); **(B)**-phosphoionsidtide 3-Kinase (PIK-domain)(bright green), **(C)**-PI3-kinase family (PI3K_rbd)(brass), **(D)**-PIK3 catalytic subunit gamma adaptor-binding domain (PIK3CG_ABD domain) (dark blue) and **(E)**-phosphatidylinositol 3 and 4-kinase (PI3_PI4 kinase domain) (red).

**FIGURE 11:**
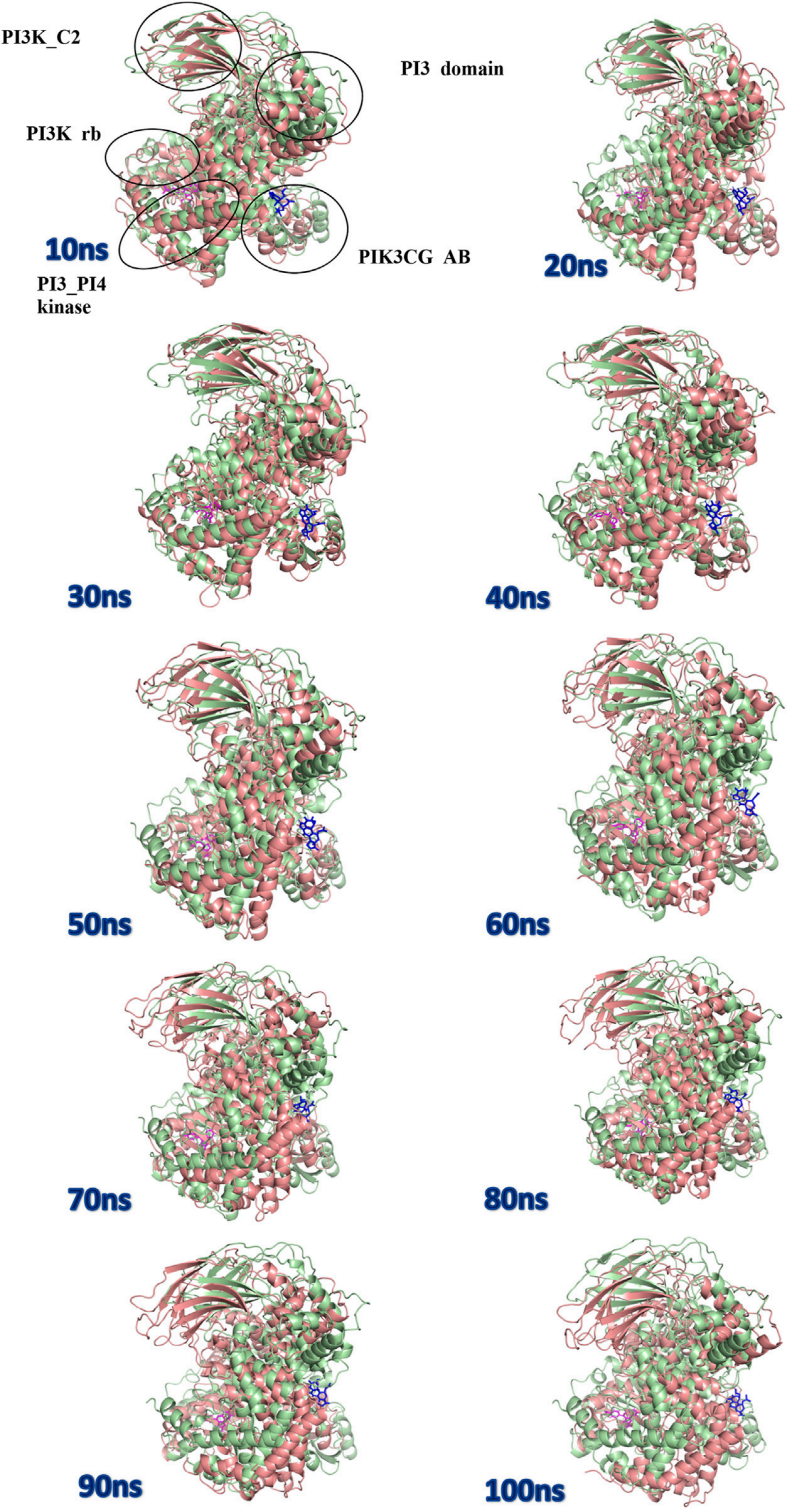
Superposition of complex structures of Wortmannin–1E7U and **1j**–1E7U after simulation for 10, 20, 30, 40, 50, 60, 70, 80, 90, and 100 ns.

**FIGURE 12:**
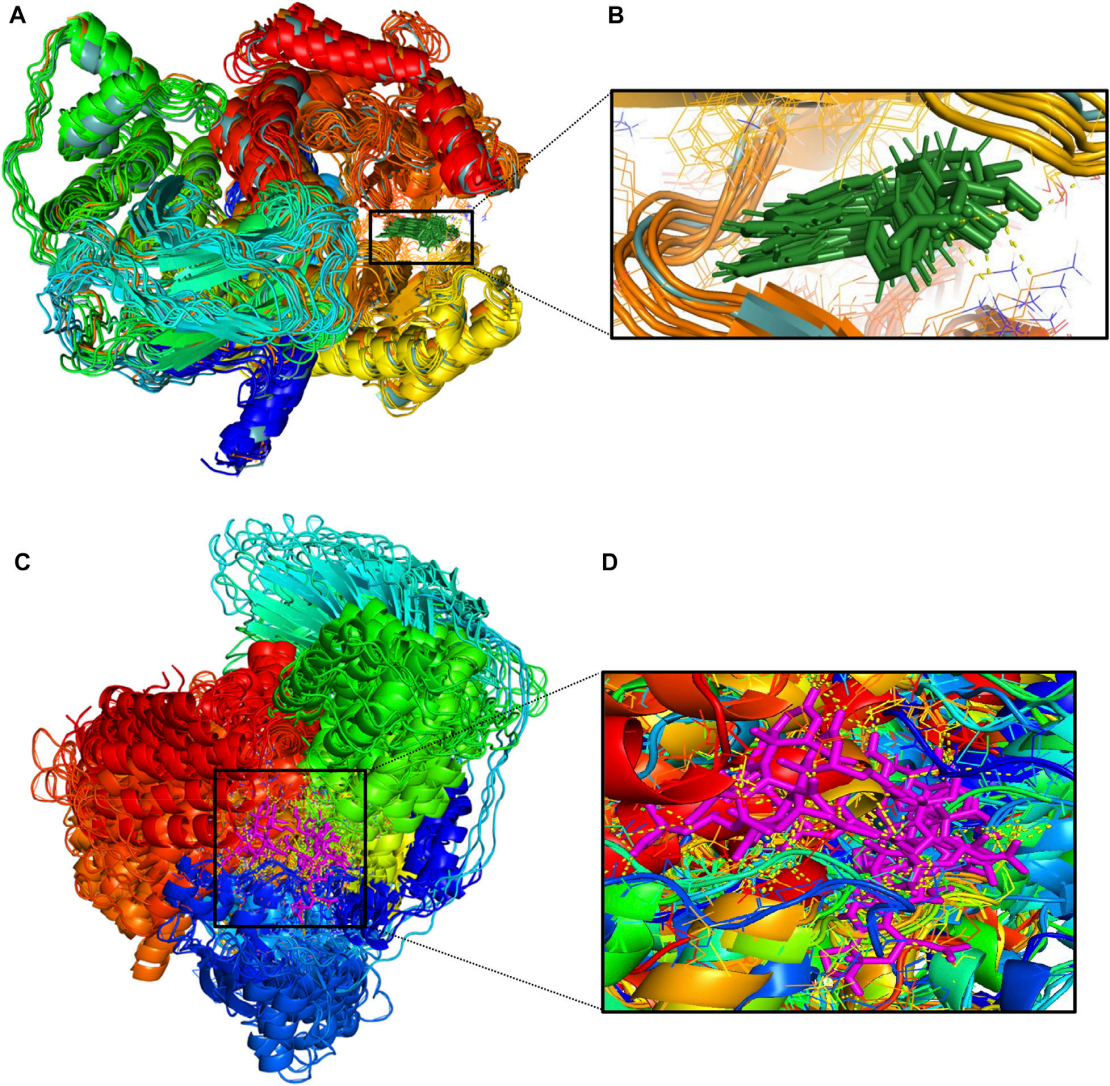
**(A)**-Conformational changes in 1E7U-**1J** (protein-ligand) complex; **(B)**-(green color for ligand) **(C)**-Conformational changes in 1E7U-Wortmannin (protein-ligand) **(D)**-(pink color for ligand) interacting with with target protien in the active site extracted from molecular dynamics trajectory at different time interims to depict the complex stability. MM-PBSA based Binding free energy Calculations.

The protein undergoes structural changes at different degrees to perform various cellular functions. Even small conformation changes can impact the functionality of the protein-ligand (Kurkcuoglu and Doruker, 2016). The protein 1E7U has five domains represented in Figure 10. Conformational changes brought up by ligands Wortmannin and **1J** in 1E7U during the 100 ns MD simulation has been depicted in Figure 10. The complex structures were extracted for every 10 ns and superimposed to examine conformational changes during the simulation. It is observed that both the ligands Wortmatnnin (red) and **1J** (green) were inducing conformational changes to the native protein 1E7U. In Figure 11, it is observed that all the five domains such as *1*) phosphoinositide 3-kinase C2 (PI3K_C2) (sky blue), *2*) phosphoionsidtide 3-Kinase (PIK-domain) (bright green), *3*) PI3-kinase family (PI3K_rbd), *4*) PIK3 catalytic subunit gamma adaptor-binding domain (PIK3CG_ABD domain) (dark blue) and *5*) phosphatidylinositol 3 and 4-kinase (PI3_PI4 kinase domain) (red) were showing minimal conformational changes. Figure 12 represents the conformation changes in the **1J**-1E7U complex throughout the simulations.

To validate the results of our study, we have carried out the MM-PBSA analysis to estimate the binding free energy of the reference and the hit complexes. Of note, shreds of literature evidence highlight that the results of MM-PBSA analysis (ΔG_bind_) correlate well with the experimental studies. (e) The inclusion of each energy component in the free energy calculations provides insight into the ligand binding mechanism. The results of MM-PBSA calculations for the standard drug (Wortmannin) and screened hit (**1j**) molecule are tabulated in Table 3. From Table 3, it is clear that the overall binding free energy of the hit molecule was −49.28 kcal mol^−1^ which is much lower than the reference molecule. Furthermore, the energy terms such as van der Waals energy, electrostatic energy, SASA energy and polar solvation energy are crucial contributors to the overall binding free energy of the complexes. In the present study, it is evident from the table that van der Waals energy and solvation energy was found to be the key driving energies that significantly contributed to the binding free energy of wortmannin and compound **1j.**

**TABLE 3:** The binding free energy of the Wortmannin and 1j complex calculated using MM-PBSA analysis.

S. No.	Compound	Binding energy	Van der Waals energy	Electrostatic Energy	Polar solvation Energy	SASA energy
1	Wortmannin	−18.15 ± 3.49	−26.84 ± 3.73	−7.25 ± 2.57	18.13 ± 4.61	−2.18 ± 0.27
2	**1j**	−49.28 ± 5.52	−62.54 ± 2.74	−17.69 ± 5.57	35.35 ± 34.73	−4.41 ± 0.26

The energy terms are measured in kcal mol^−1^.

### ADME screening

In the present investigation, the drug ability of the small molecules were screened using Absorption Distribution Metabolism and Excretion properties incorporated in QikProp module of Schrödinger suite. The important descriptors such as stars, HOA and Lipinski’s rule of five were subjected for considerations. The star value describes the violations of the descriptors by comparing with 95% of known inhibitors. The HOA highlights the human oral absorption, where the compound with HOA >2 was identified as efficient drug molecule. Further, the Lipinski’s rule of five includes molecular weight (<500), hydrogen bond acceptor (HBA≤ 10), donor (HBD ≤5), and octanol/water coefficient (QPlogPo/W < 5). The results from ADME analysis were illustrated in Table 4. Of note, compound **1j** was found to satisfy the above-mentioned criteria and therefore identified as hit compound for further analysis.

**TABLE 4:** ADME properties of the reference and the hit compounds.

											
Compound ID	Mol. Wt	Stars	CNS	Human oral absorption	Donor HB	Accpt HB	QPlogPoct	QPlogPo/w	QPlogS	QPlogBB	QPlogKp
Wortmannin	428.438	0	−1	2	0	11.2	19.124	0.359	−1.178	−0.981	−4.059
1a	331.543	4	2	3	2	5	18.118	2.669	−2.117	1.272	−5.823
1b	347.543	2	2	2	2	6.7	18.885	1.907	−1.177	1.101	−5.968
1c	407.595	2	2	2	2	10.1	22.309	1.441	−0.813	0.953	−5.82
1d	346.558	1	2	2	4	6	21.311	1.104	0.14	0.779	−6.409
1e	333.516	1	2	2	3	6.7	19.691	1.185	−0.727	0.825	−6.565
1f	331.543	2	2	3	2	5	17.941	2.433	−1.948	1.122	−6.129
1g	361.57	3	2	3	2	6.7	19.823	2.419	−1.905	1.208	−5.732
1h	361.57	4	2	3	2	6.7	19.82	2.48	−2.094	1.251	−5.644
1i	421.622	3	2	3	2	10.1	23.294	2.117	−1.967	1.148	−5.415
1j	378.557	2	1	2	4	6	21.842	1.585	−1.136	0.347	−7.087
1k	331.543	3	2	3	2	5	18.278	2.725	−2.27	1.263	−5.838
1l	345.57	2	2	3	2	5	18.783	2.747	−2.402	1.059	−6.246
1m	361.57	2	2	3	2	6.7	19.762	2.254	−1.826	1.044	−6.061
1n	421.622	2	2	3	2	10.1	23.406	2.098	−1.912	1.078	−5.545
1o	410.439	2	2	3	2	5	18.973	2.916	−2.543	1.254	−6.142
1p	353.498	3	2	3	2	5	18.147	2.781	−2.025	1.48	−5.798

### Anticancer activity-prediction of activity spectra for substance

The anti-cancer activity of the compound was further assessed by employing PASS prediction algorithm and the results are given in Table 5. It is evident from the table that the probability of actives was higher when compared with the probability of inactive ones among all the investigated compounds. Among the studied compounds, the compound such as **1p**, and **1j**, exhibit higher active scores (greater than 0.5). This indicates that there is a greater possibility of these compounds to have experimental activity (Stepanchikova et al., 2003). Additionally, all these compounds were predicted to have anti-cancerous activity including breast cancer.

**TABLE 5:** Prediction of biological activity using PASS algorithm.

S. No.	Compound ID	Pa	Pi	Activity
1	**1a**	0.423	0.096	Antineoplastic
2	**1b**	0.177	0.110	Antineoplastic (breast cancer)
3	**1c**	0.274	0.063	Antineoplastic (breast cancer)
4	**1d**	0.400	0.104	Antineoplastic
5	**1e**	0.167	0.120	Antineoplastic (breast cancer)
6	**1f**	0.367	0.118	Antineoplastic
7	**1g**	0.437	0.091	Antineoplastic
8	**1h**	0.396	0.105	Antineoplastic
9	**1i**	0.211	0.088	Antineoplastic (breast cancer)
10	**1j**	0.509	0.006	Antineoplastic alkaloid
11	**1k**	0.410	0.101	Antineoplastic
12	**1l**	0.389	0.108	Antineoplastic
13	**1m**	0.430	0.093	Antineoplastic
14	**1n**	0.230	0.080	Antineoplastic (breast cancer)
15	**1o**	0.370	0.116	Antineoplastic
16	**1p**	0.594	0.046	Antineoplastic

## Conclusions

Herein we have reported the potential application of a series of synthesized pyrido fused imidazo[4,5-c]quinoline derivatives (Rao and Chanda, 2021) as anti-tumor drug candidates *via in silico* evaluation. The initial pharmacokinetic and pharmacodynamics evaluations have revealed the drug likeliness of all the proposed candidates. Further investigation on their potential as drug-like candidate was evaluated based on their ability to inhibit the PI3K family of enzymes *via in silico* evaluation. Based on the scores obtained from docking investigations **1j** was identified to exhibit the highest activity through its binding interactions with the active site residues of PI3K enzyme. The energetics associated with static interactions also revealed **1j** as the most potential candidate while the dynamic investigations including RMSD, RMSF, Rg, SASA and hydrogen bonding also supported the same through relative stabilization induced through ligand interactions. It can be concluded that the presence of a strong electron-withdrawing nitro group at the para position of the attached phenyl ring of **1j** could have played a key role in the observed activity. Molecular dynamic simulations have revealed average stabilization energy of −498.24 × 10^3^ kcal mol^−1^ upon interaction with PI3K. Moreover, the RMS fluctuations indicate the interacting residues as ASN951, ASP864, THR887, LYS890, THR886, TRP812, GLU880, ILE 881, ASN 951 and ILE963, while the hydrogen bond analysis revealed the presence of seven hydrogen bonds. All the above evaluations revealed the highest potency of **1j** as anticancer drug. Further evaluations with PASS prediction algorithm also supported the above results as promising anticancer agents. Therefore, we propose that the possibility of these candidates as potential therapeutic agents can further be tuned by modulating their molecular structures by adding a variety of electron donating or accepting moieties appropriately in the imidazo quinoline backbone to possess enhanced biological activities, which could be made possible through detailed electronic structure evaluations. *In vitro* and *in vivo* evaluations may further be needed to support the above claims. The same procedure may further be implemented with other fused heterocyclic to enrich the library of available anti-cancer candidates.

## Data Availability

The original contributions presented in the study are included in the article/Supplementary Material, further inquiries can be directed to the corresponding author.
