# Supplementary material for: In-silico molecular modelling, MM/GBSA binding free energy and molecular dynamics simulation study of novel pyrido fused imidazo[4,5-c]quinolines as potential anti-tumor agents
Source: Front Chem. 2022 Sep 30;10:991369. doi: 10.3389/fchem.2022.991369 (PMC9566731; doi:10.3389/fchem.2022.991369)
Supplement: Supplementary file 1 [file DataSheet1.docx]

**Supporting Information**

***In-silico* Molecular Modelling, MM/GBSA Binding Free Energy and Molecular Dynamics Simulation Study of Novel Pyrido fused imidazo[4,5-*c*]quinolines as Potential Anti-tumor Agents**

Upala Dasmahapatra,^a#^ Chitluri Kiran Kumar^c#^ Soumyadip Das,^a^ Prathima Thimma Subramanian,^b^, Poornimaa Murali^c^, Arnold Emerson Isaac^c^, Karuppasamy Ramanathan,^c^ Balamurali MM,^b*^ Kaushik Chanda^a*^

*^a^*Department of Chemistry, School of Advanced Sciences, Vellore Institute of Technology, Vellore - 632 014, Tamil Nadu, India

*^b^* Division of Chemistry, School of Advanced Sciences, Vellore Institute of Technology, Chennai campus, Vandalur-Kelambakkam Road, Chennai - 600 127, Tamil Nadu, India

*^c^*Department of Biotechnology, School of BioSciences and Technology, Vellore Institute of

Technology, Vellore, Tamil Nadu, India, 632014

E-mail: [chandakaushik1@gmail.com](mailto:chandakaushik1@gmail.com)

[mmbala@gmail.com](mailto:mmbala@gmail.com)

**Contents**

**Autodock and Glide images of all compounds………………………S2-S26**

| **Entry** | **3D stick representation** | **3D Ribbon representation** |
| --- | --- | --- |
| **1a** | 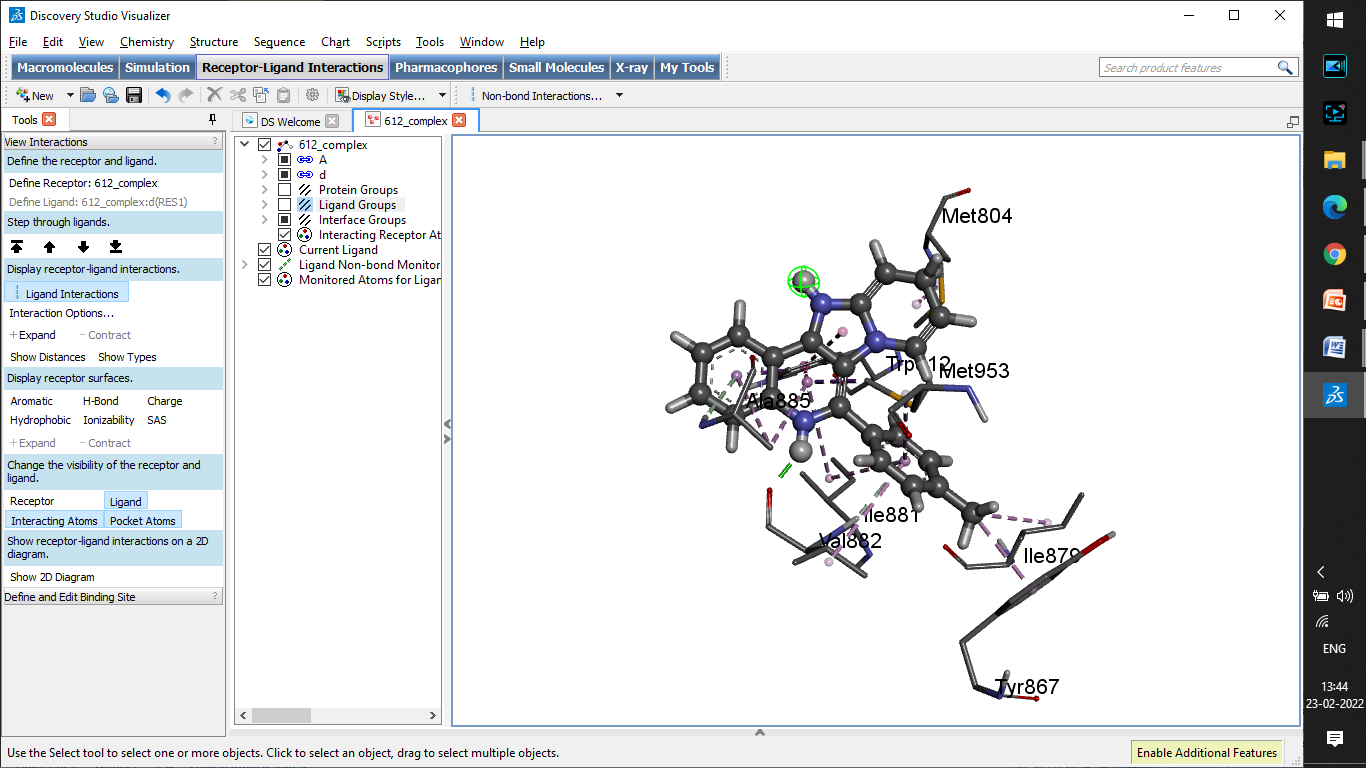 | 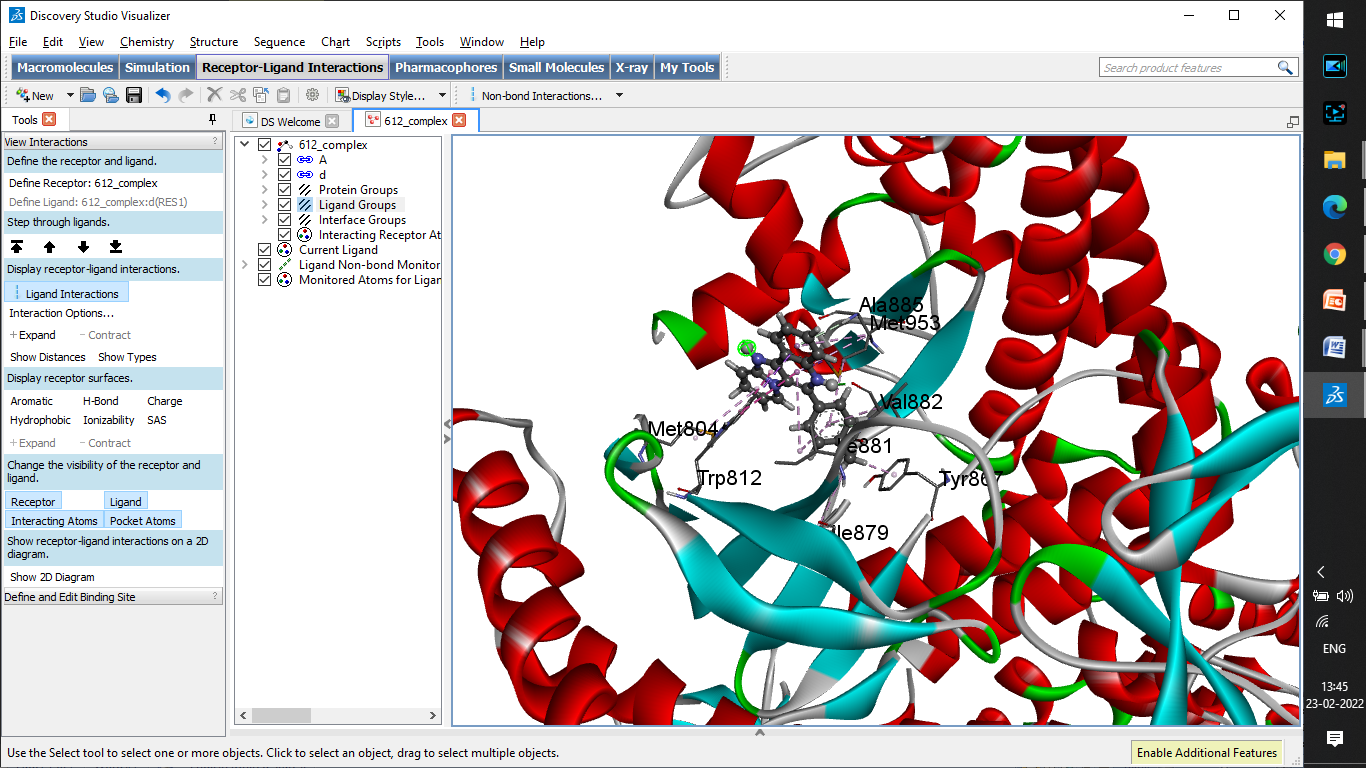 |
| **1b** | 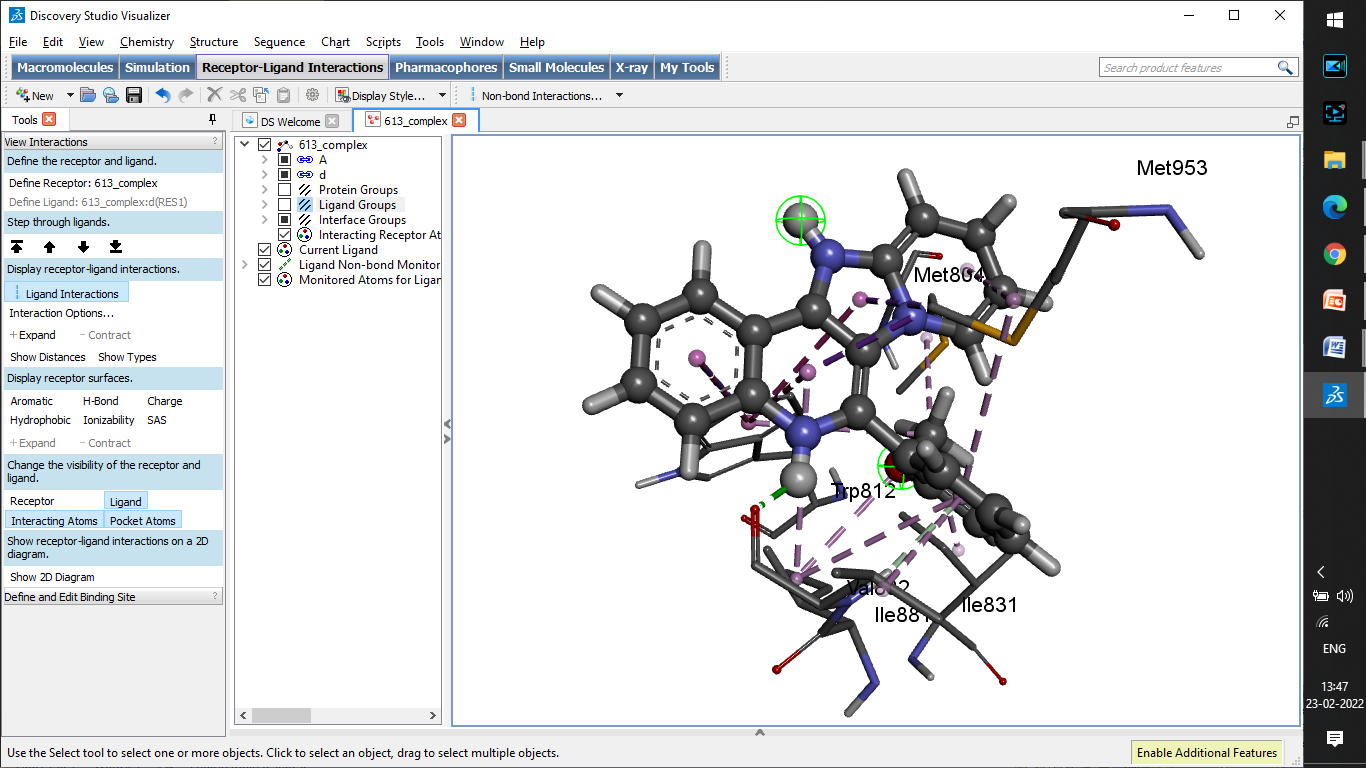 | 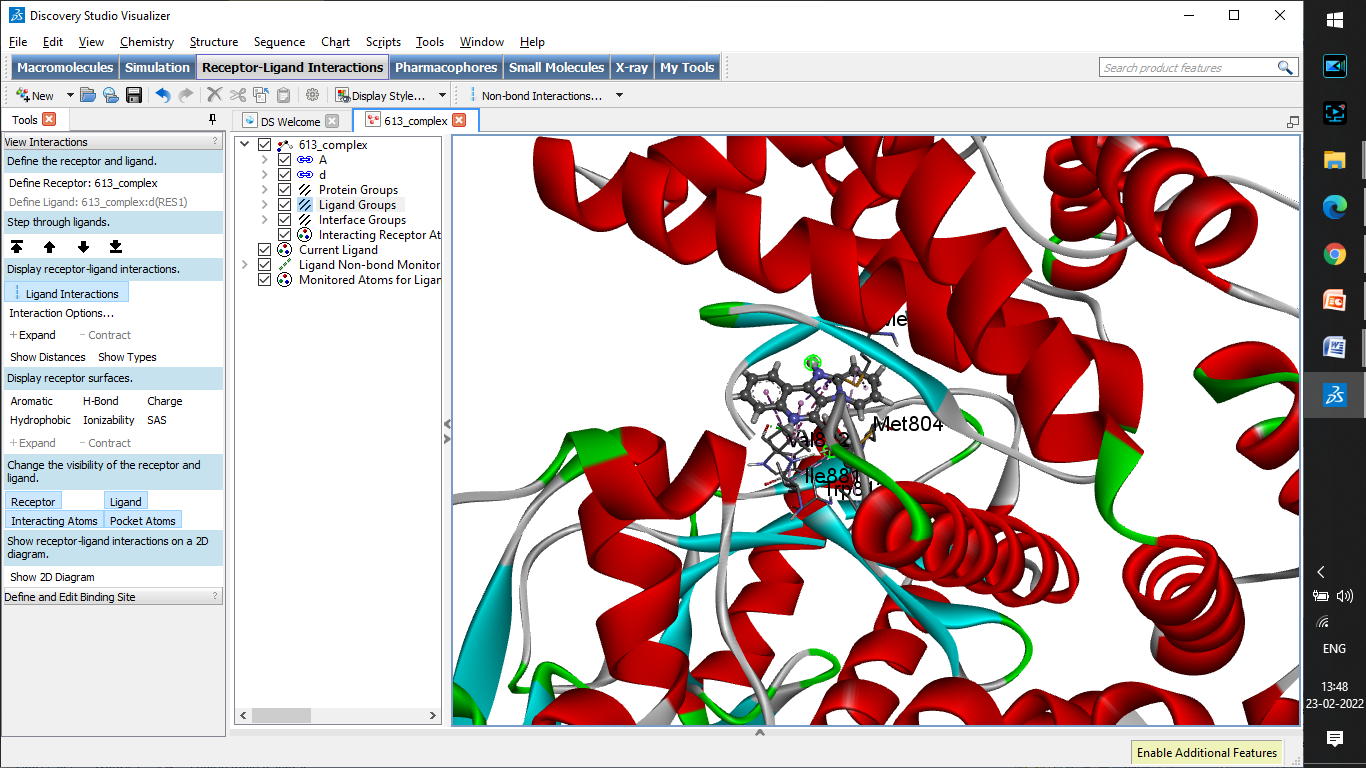 |
| **1c** | 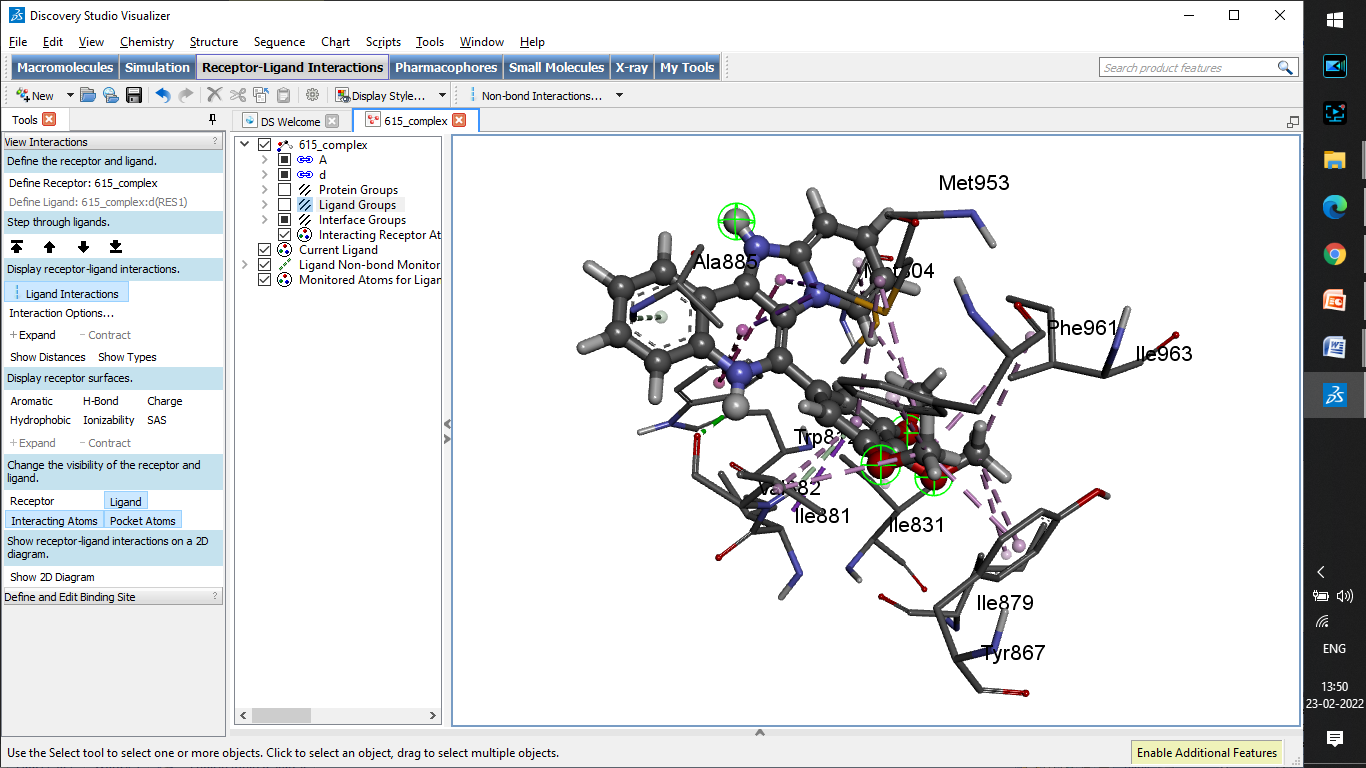 | 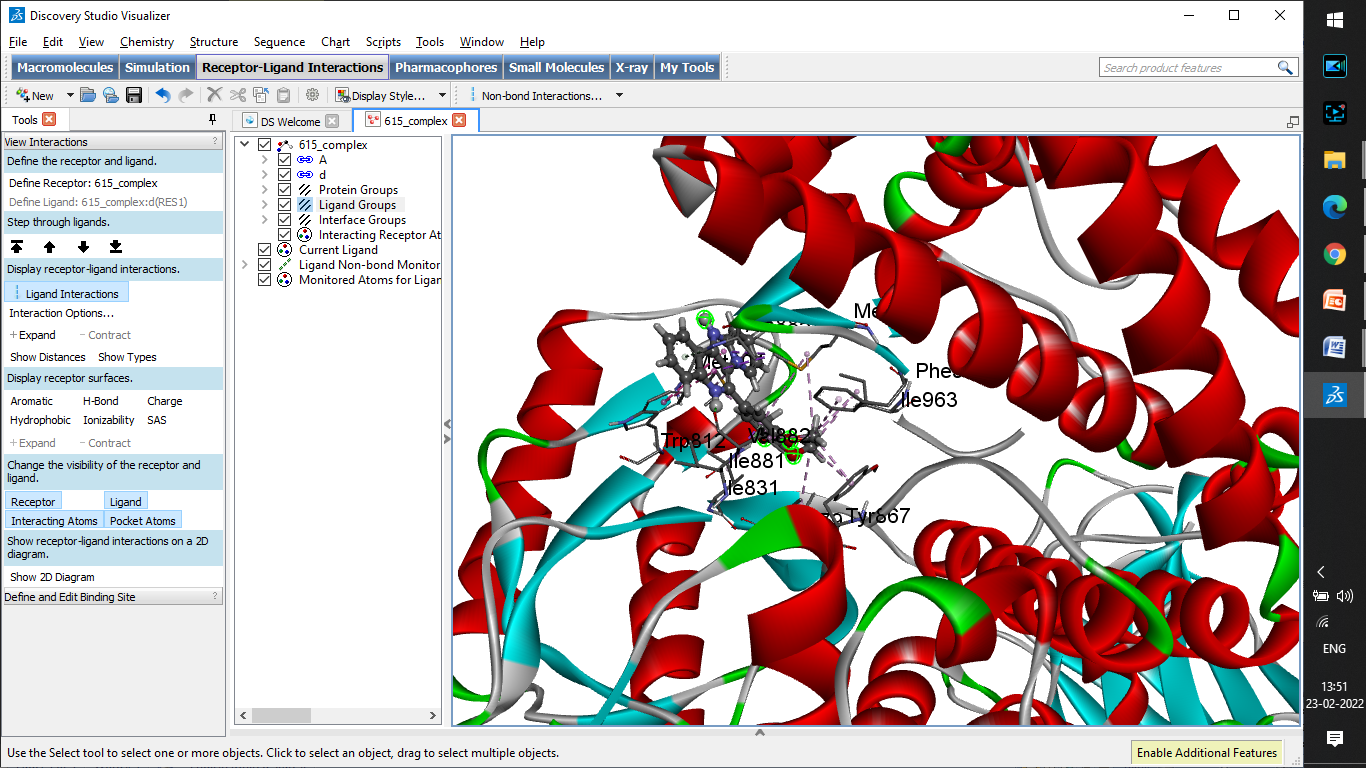 |
| **1d** | 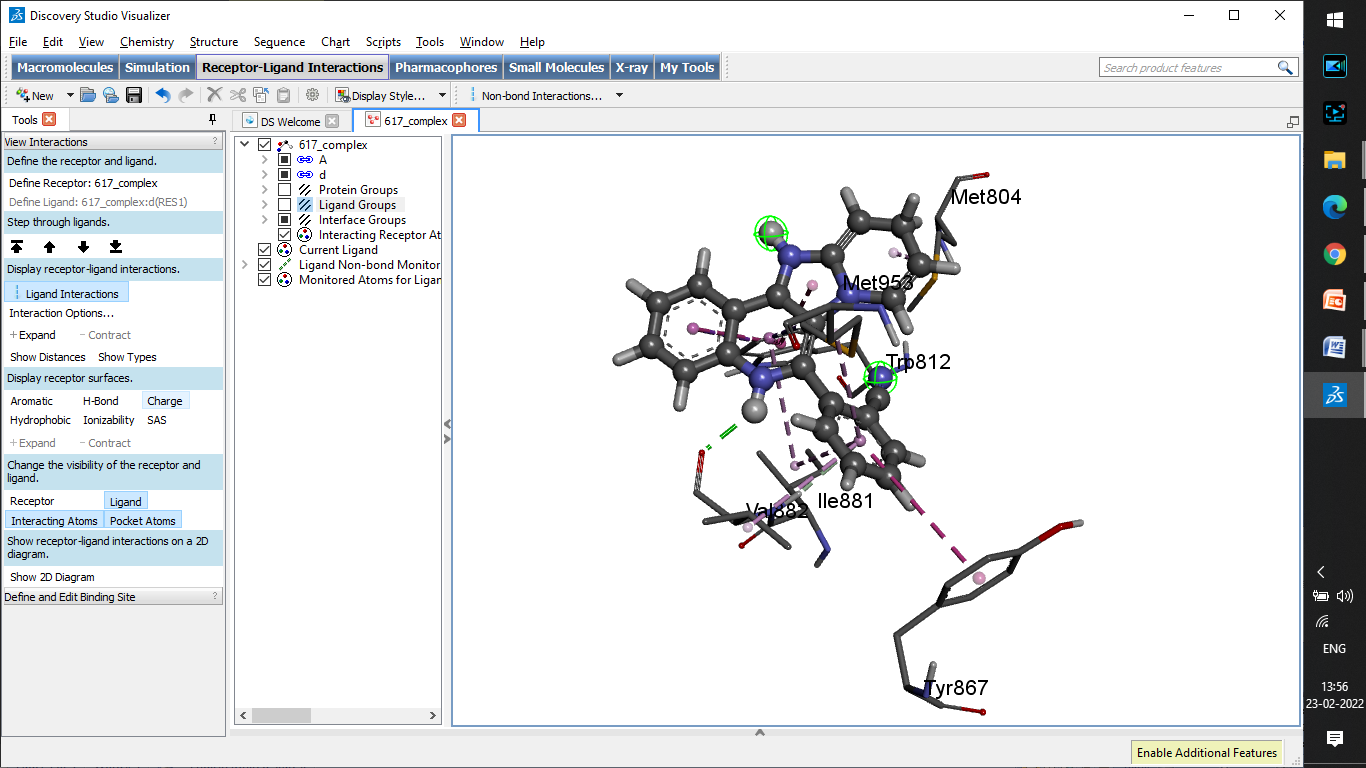 | 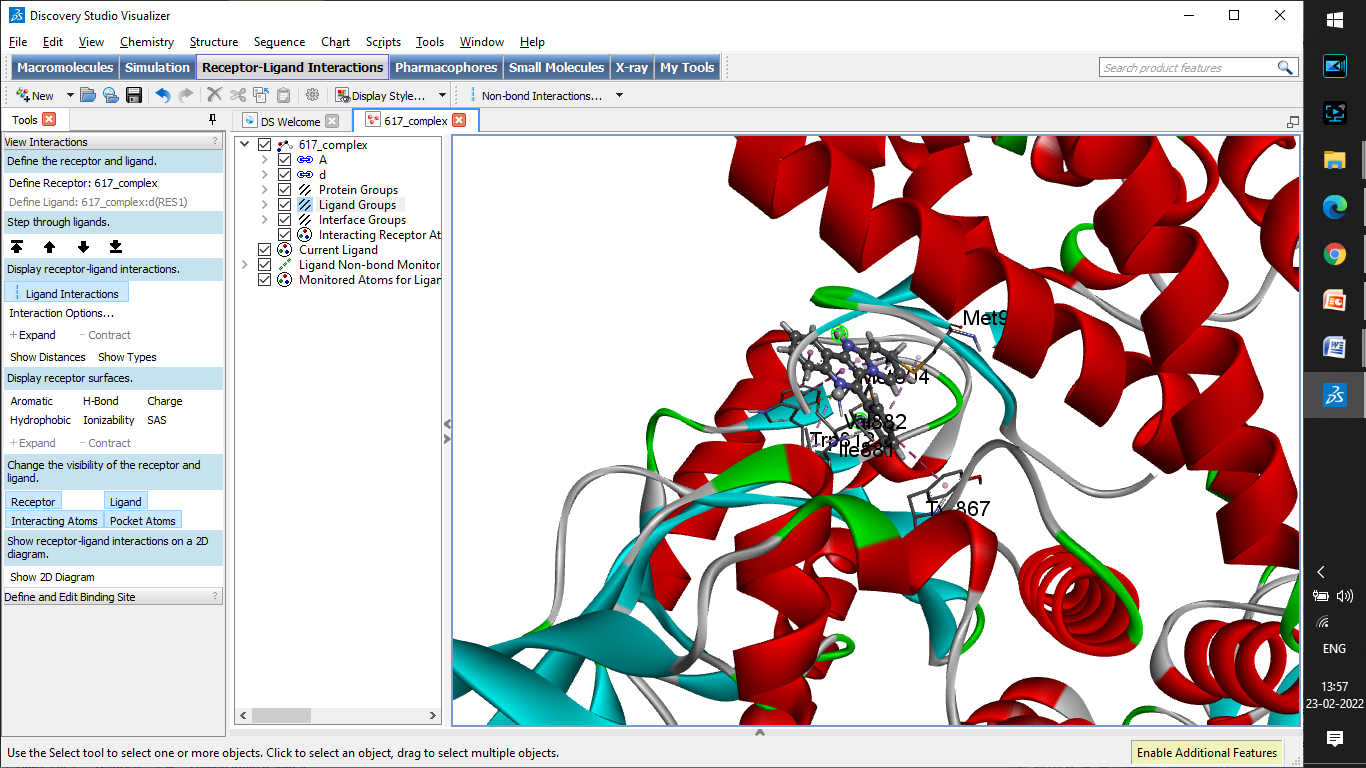 |
| **1e** | 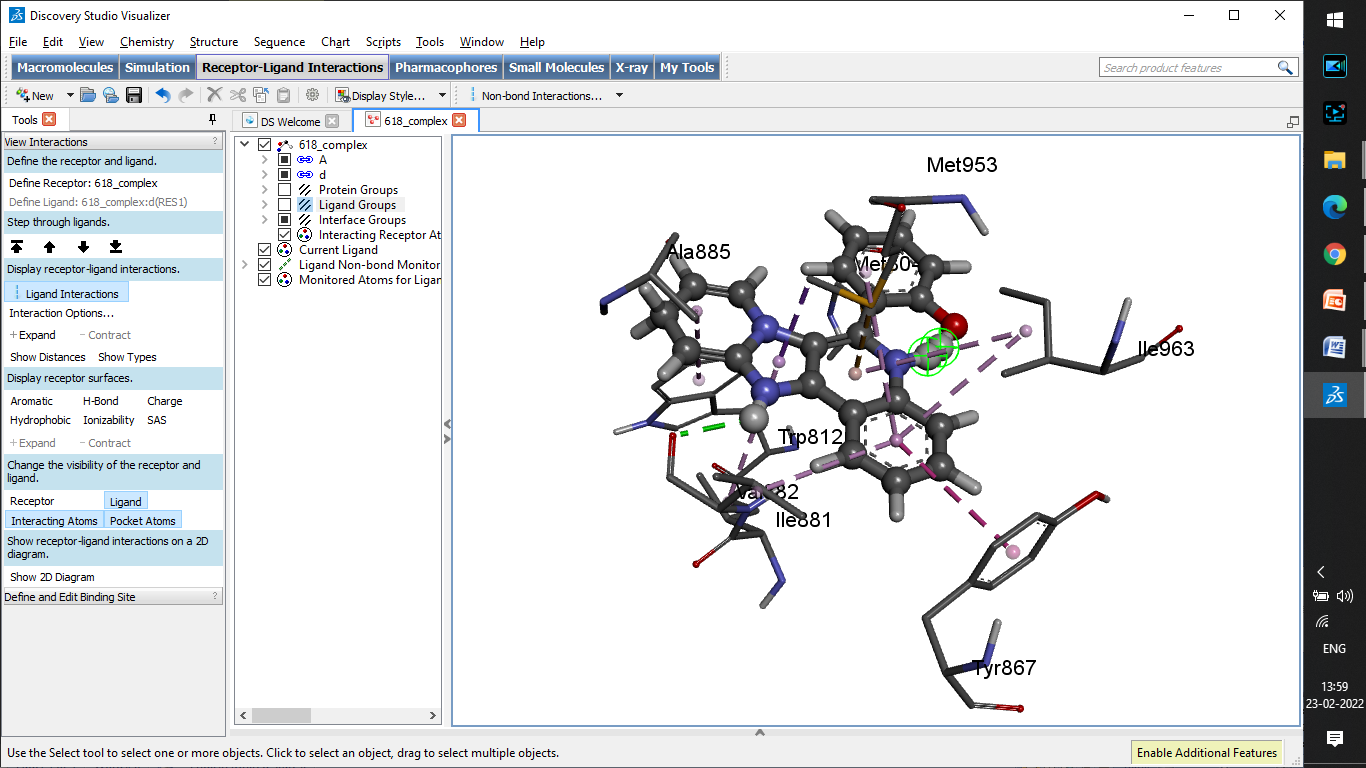 | 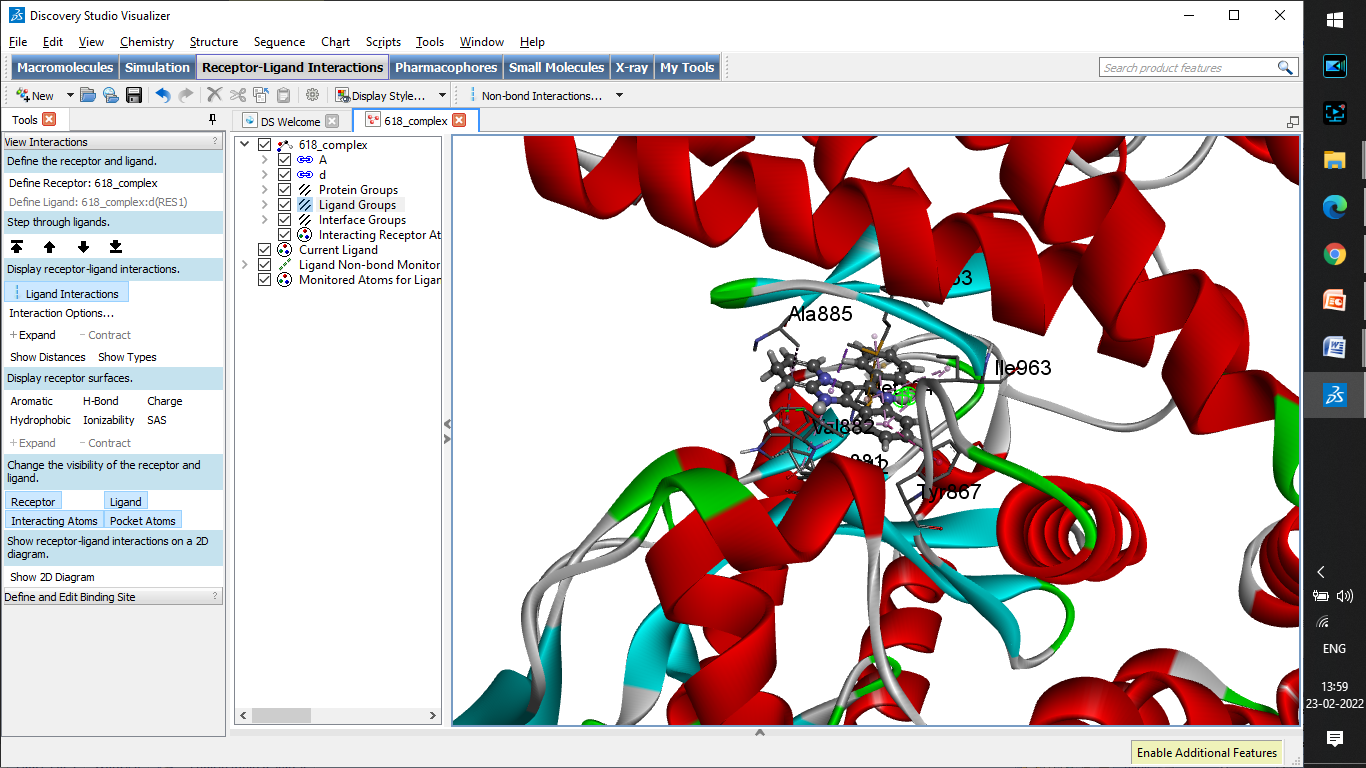 |
| **1f** | 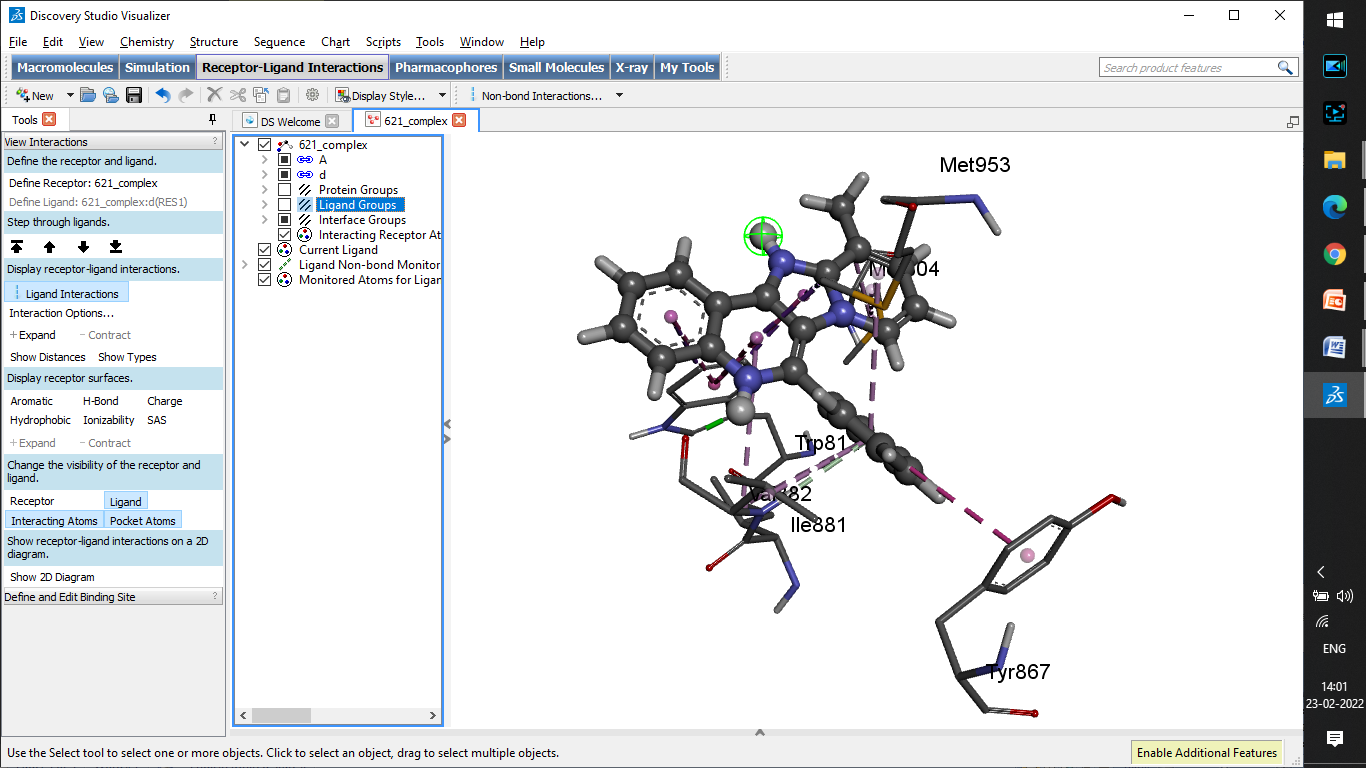 | 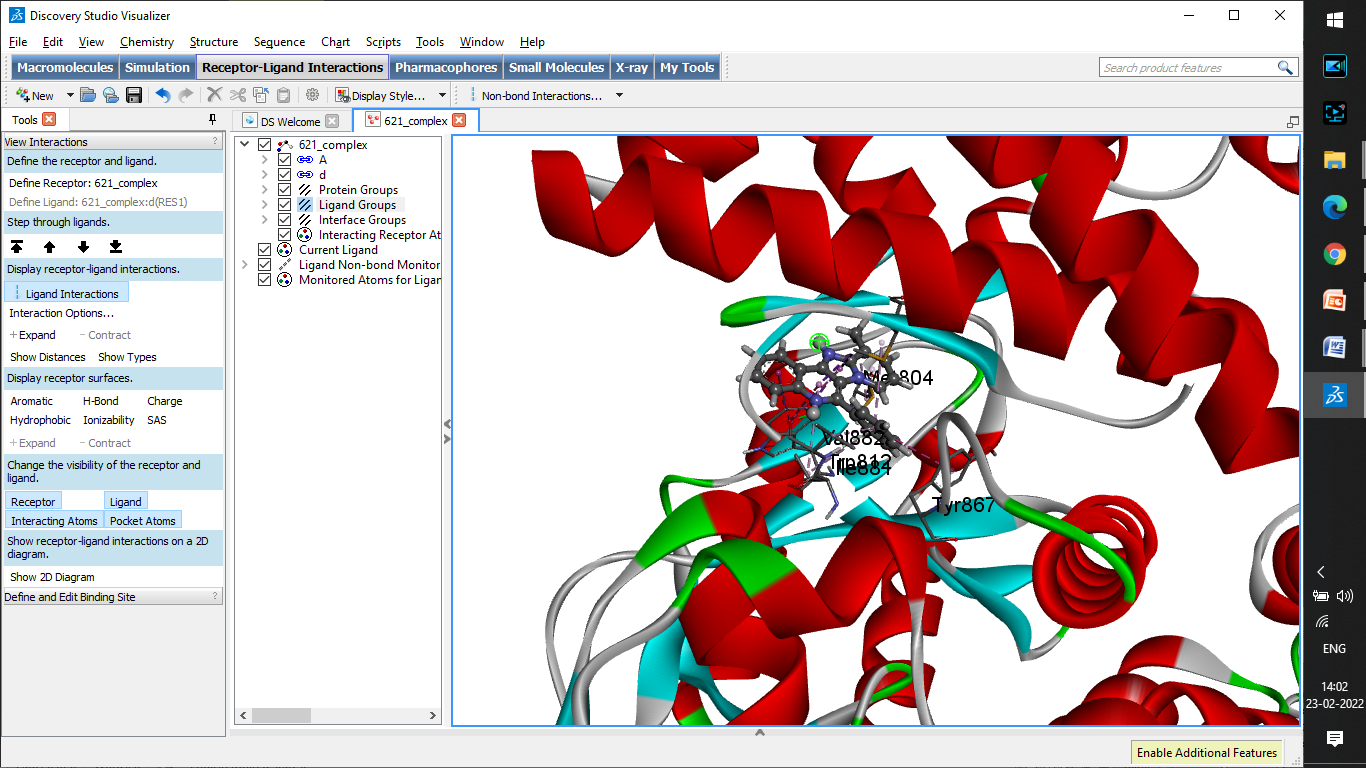 |
| **1g** | 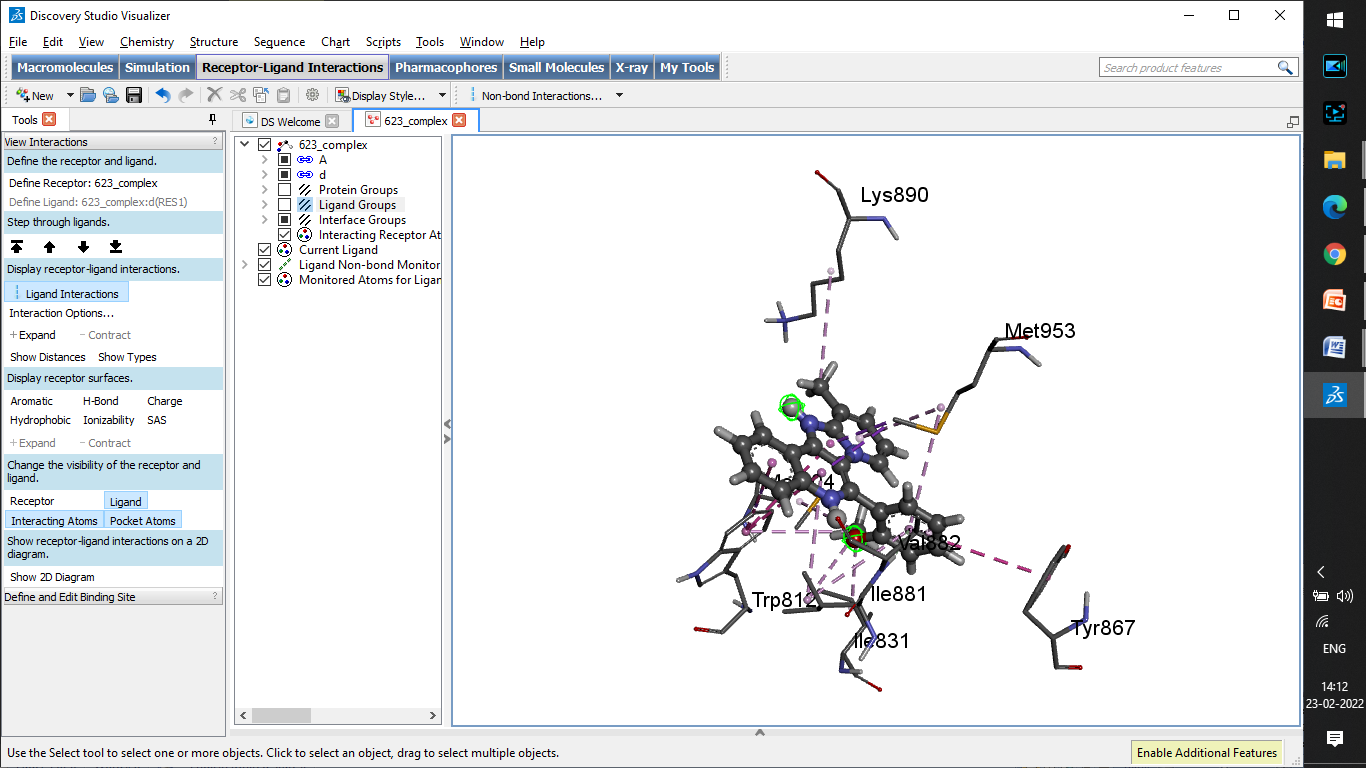 | 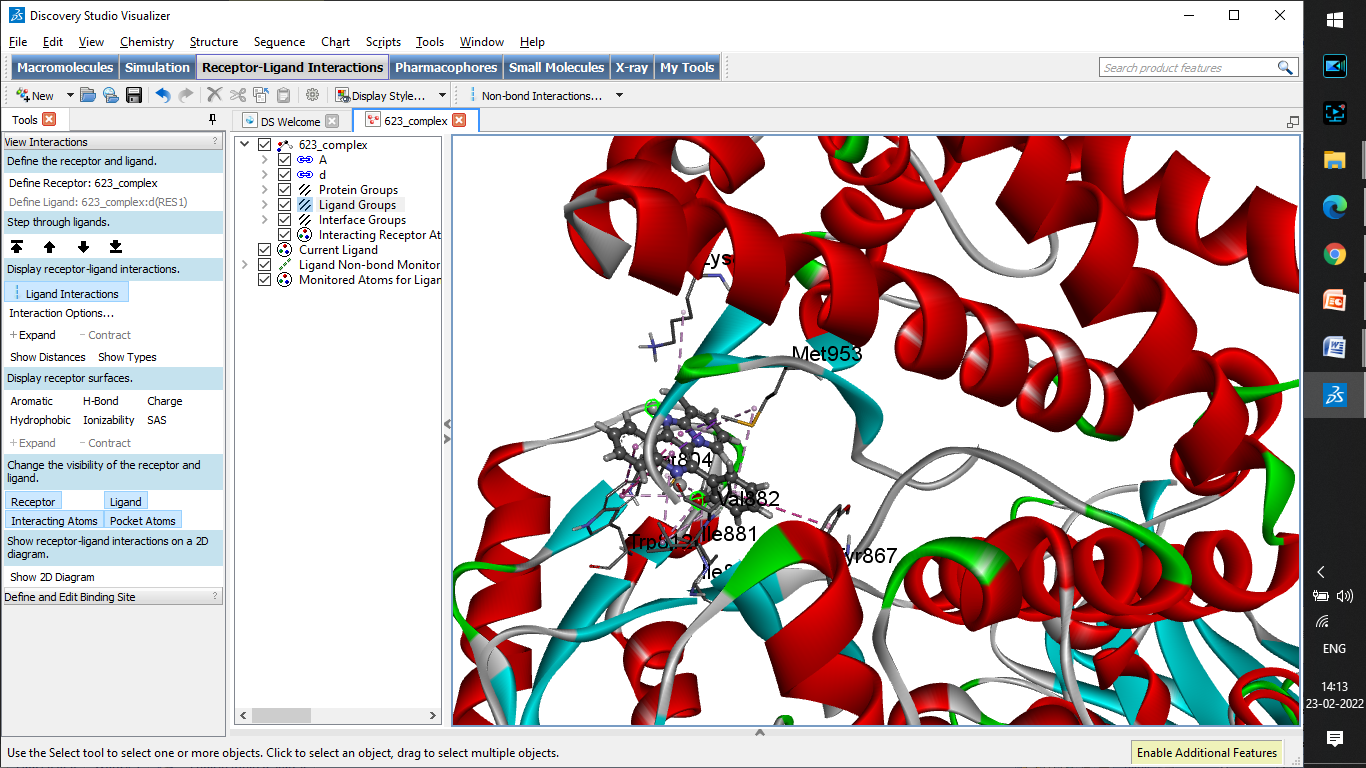 |
| **1h** | 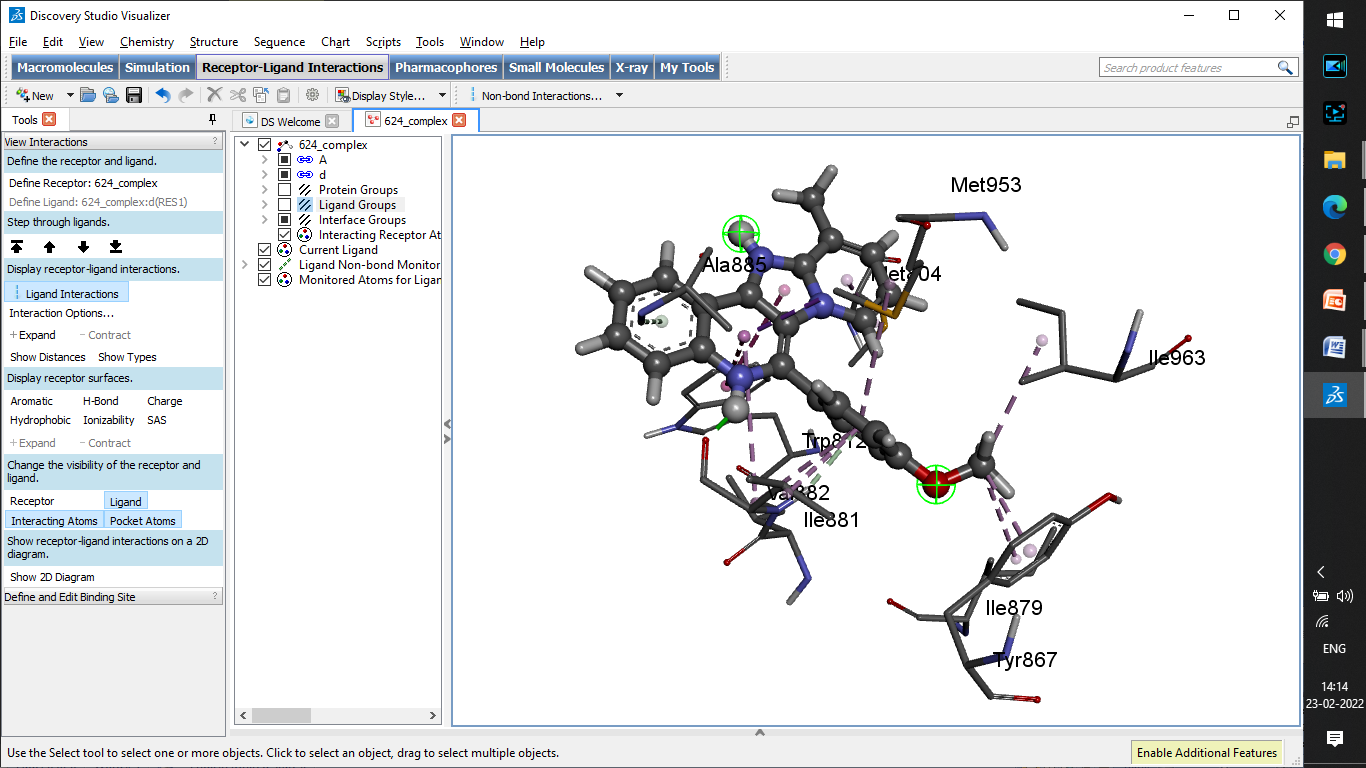 | 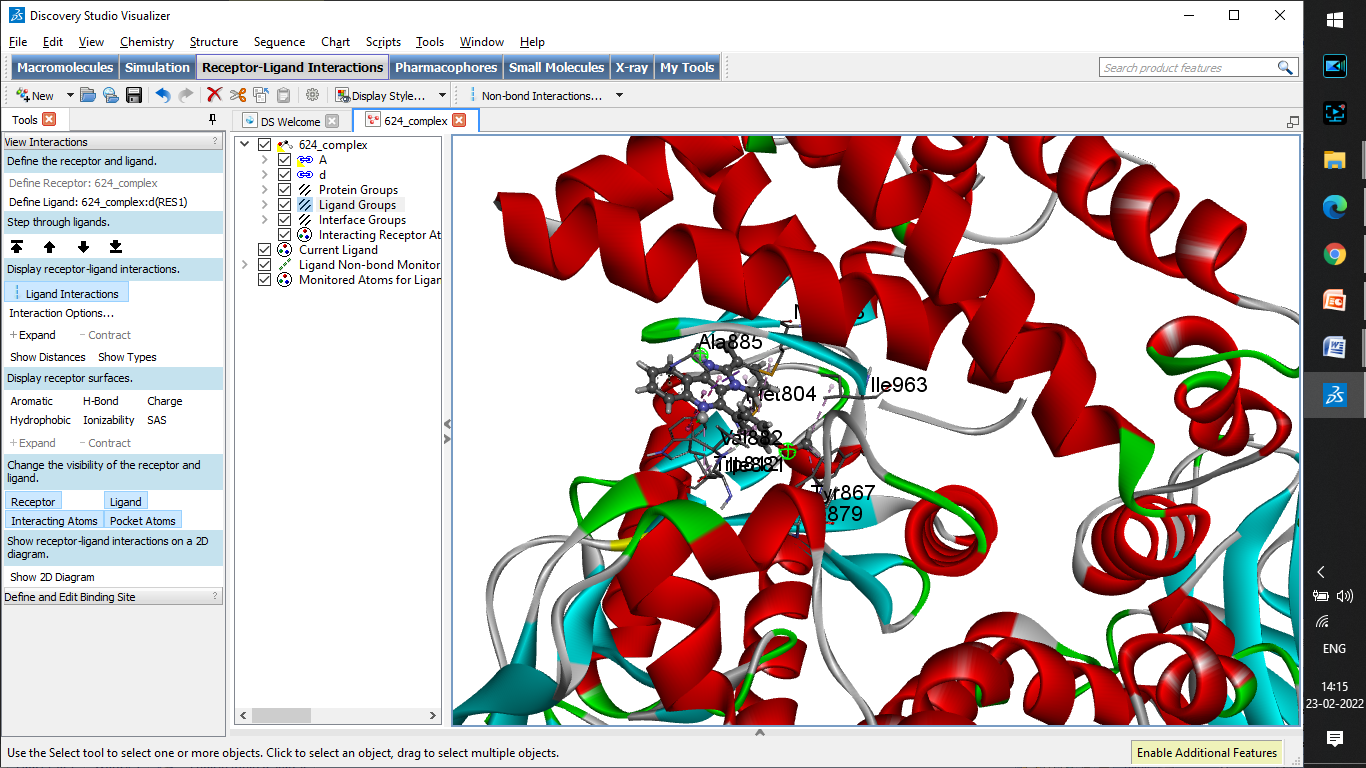 |
| **1i** | 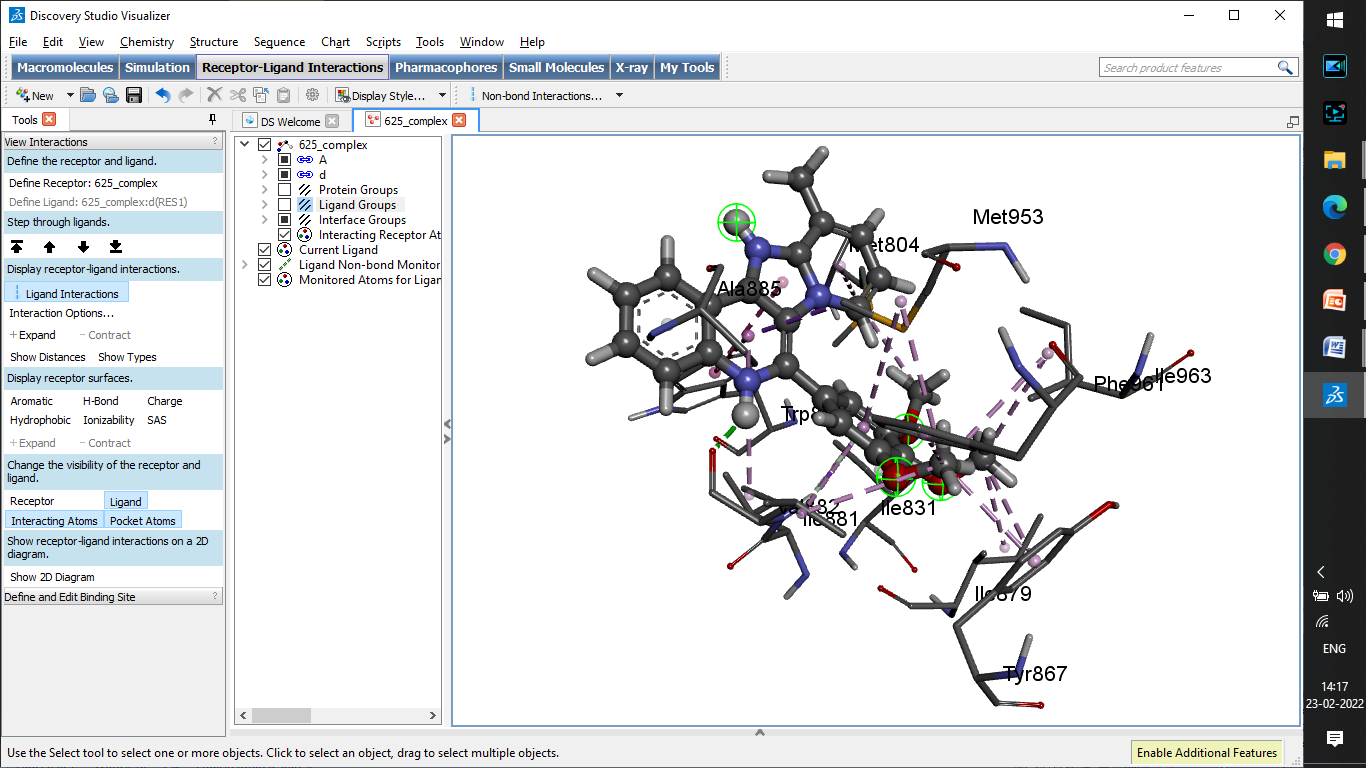 | 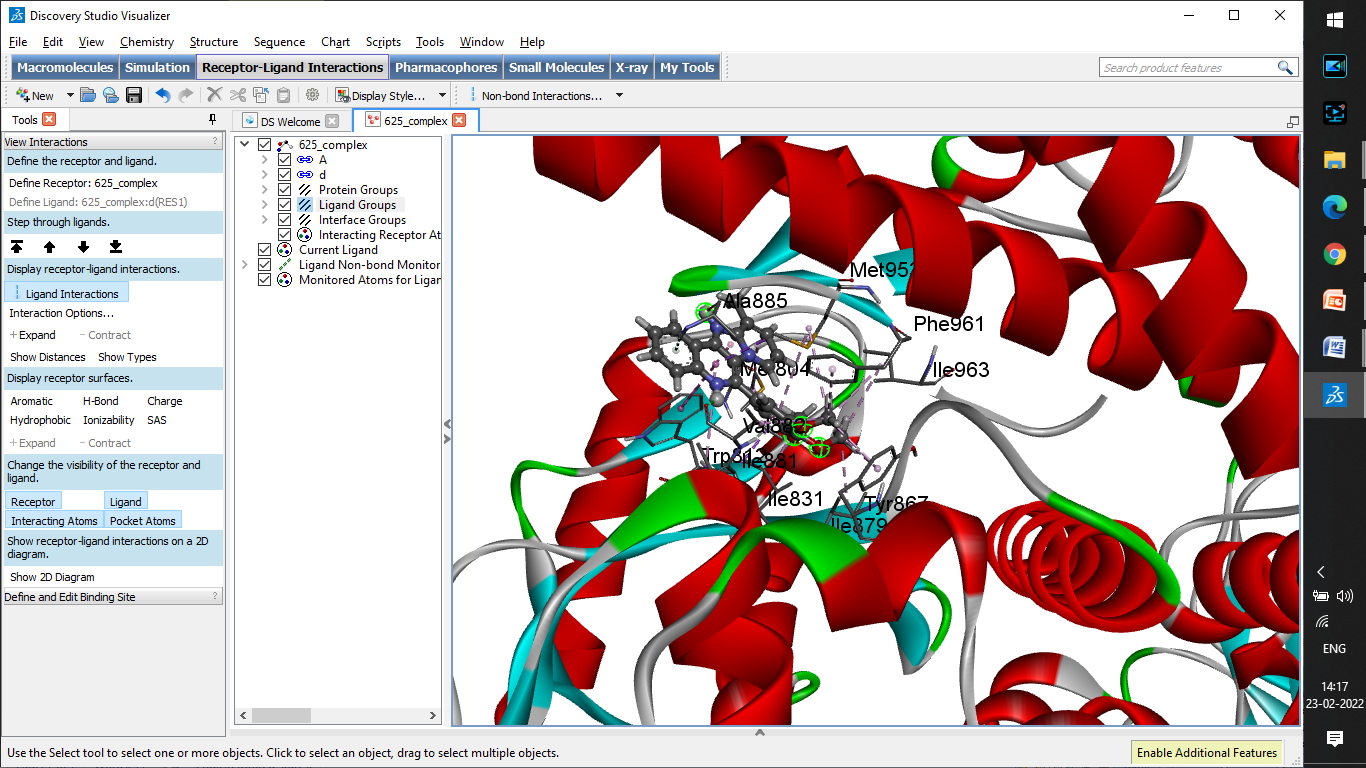 |
| **1k** | 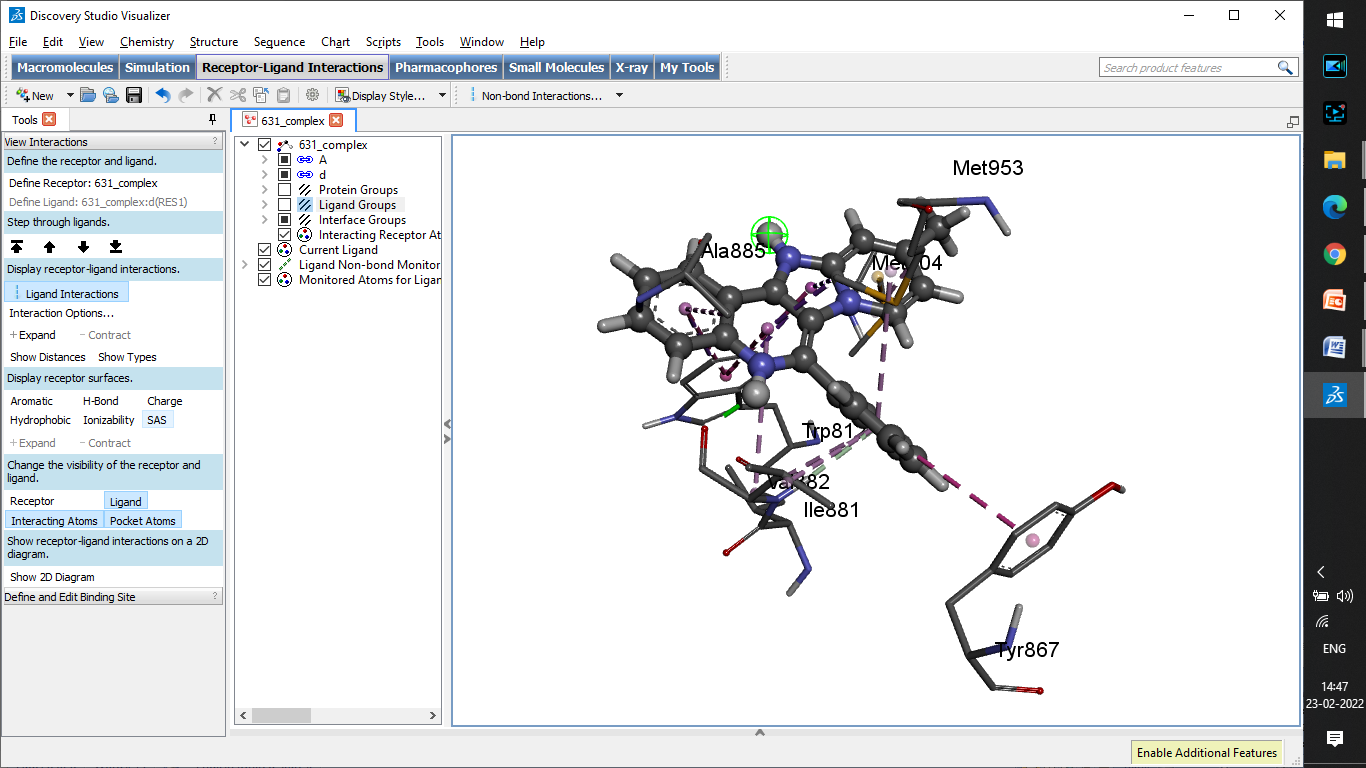 | 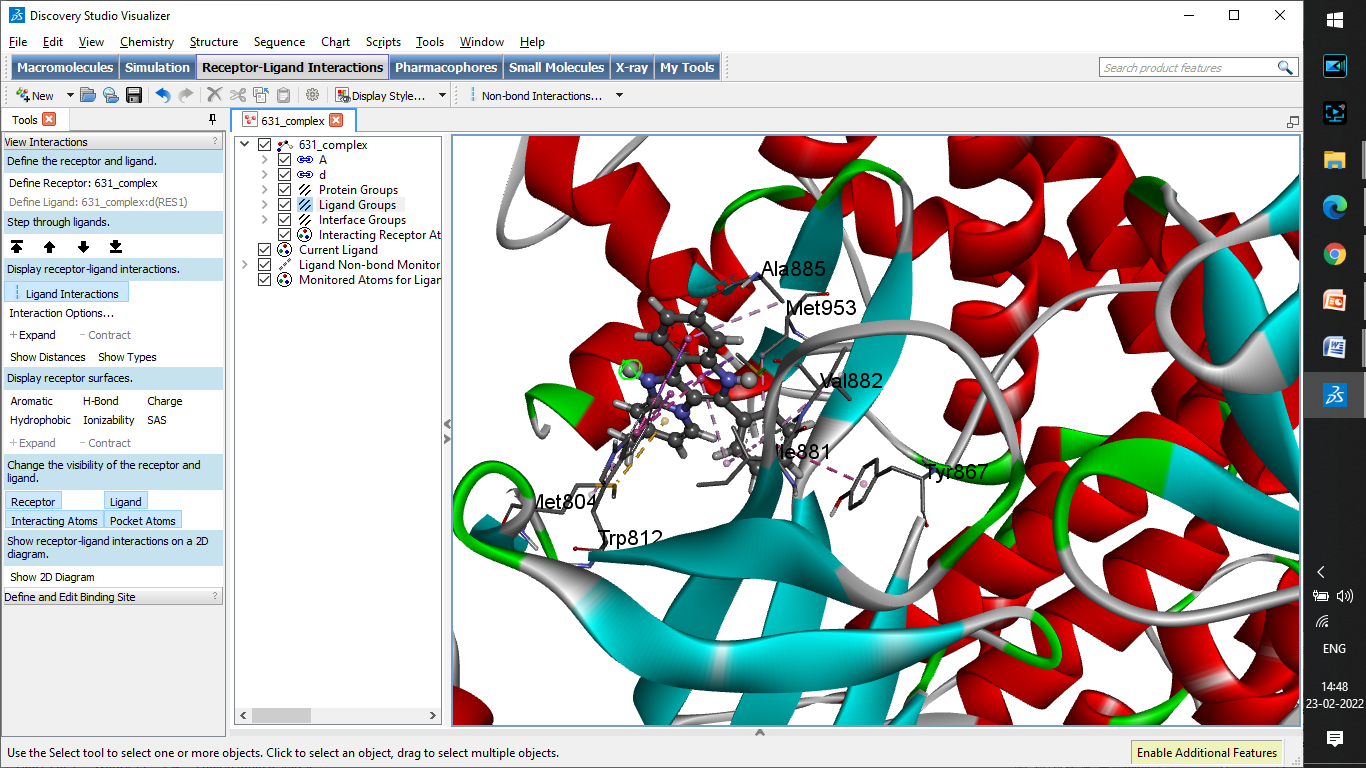 |
| **1l** | 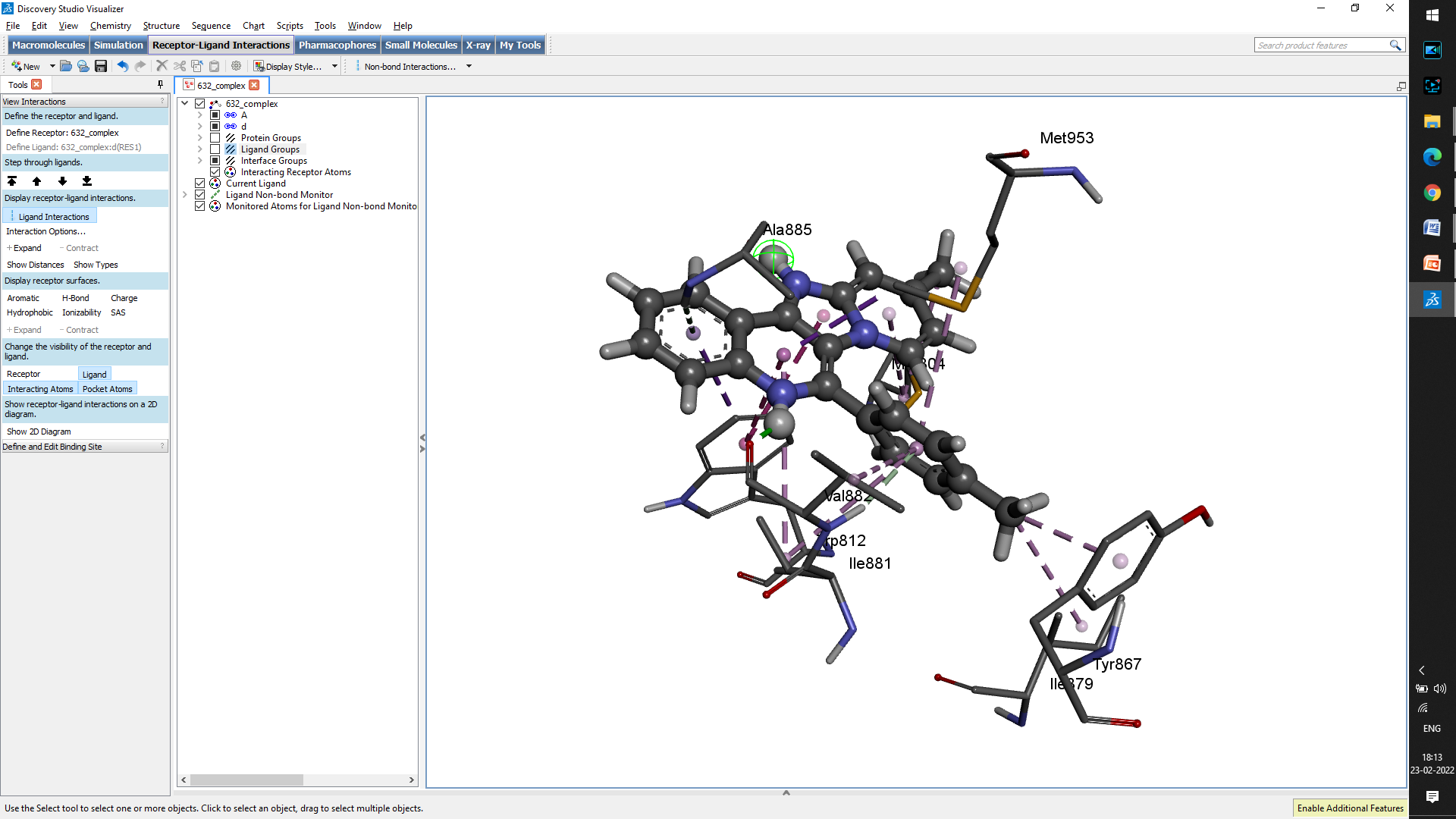 | 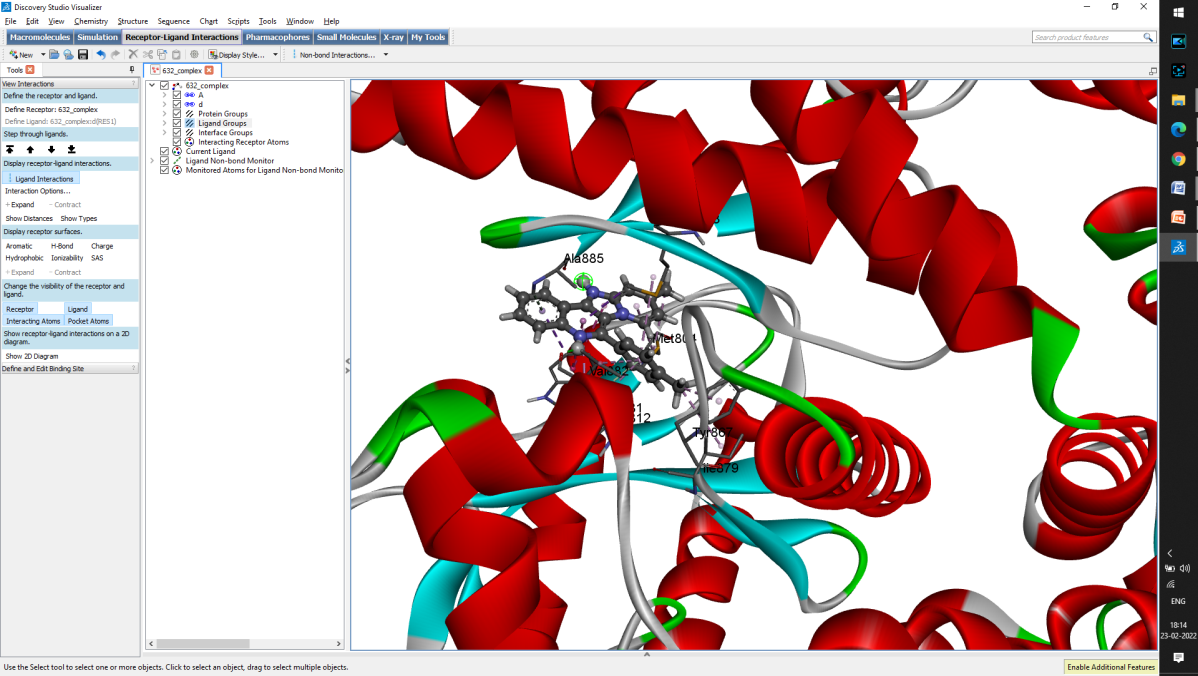 |
| **1m** | 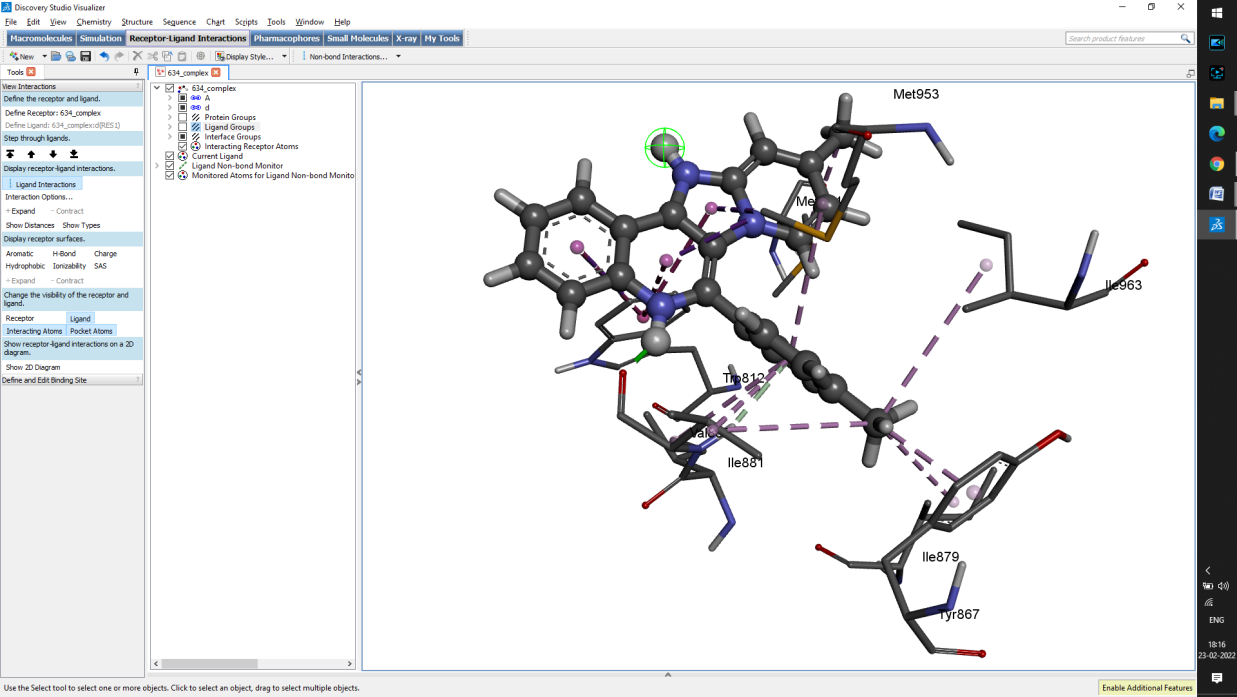 | 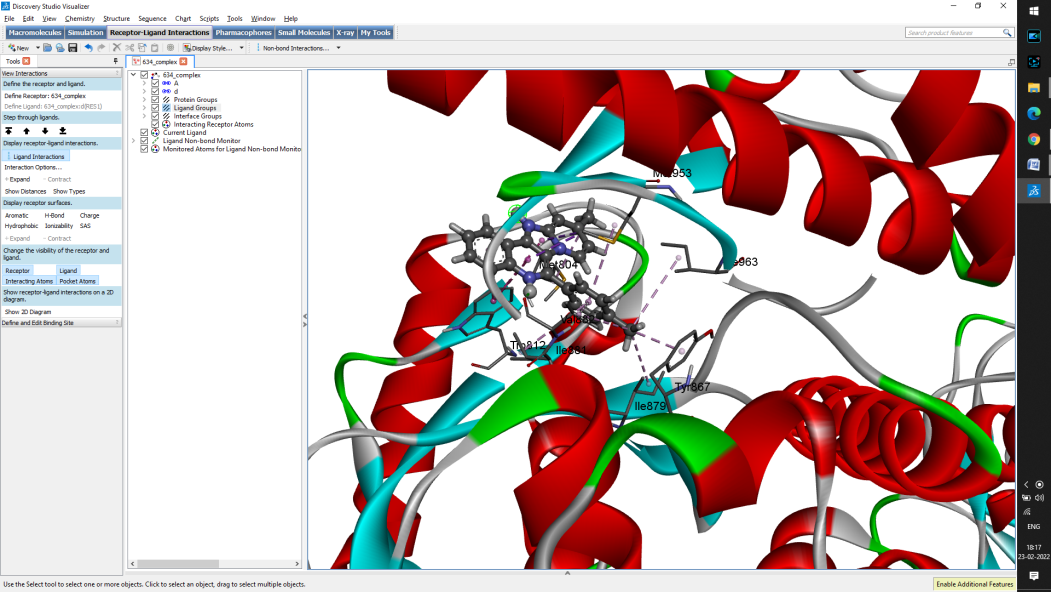 |
| **1n** | 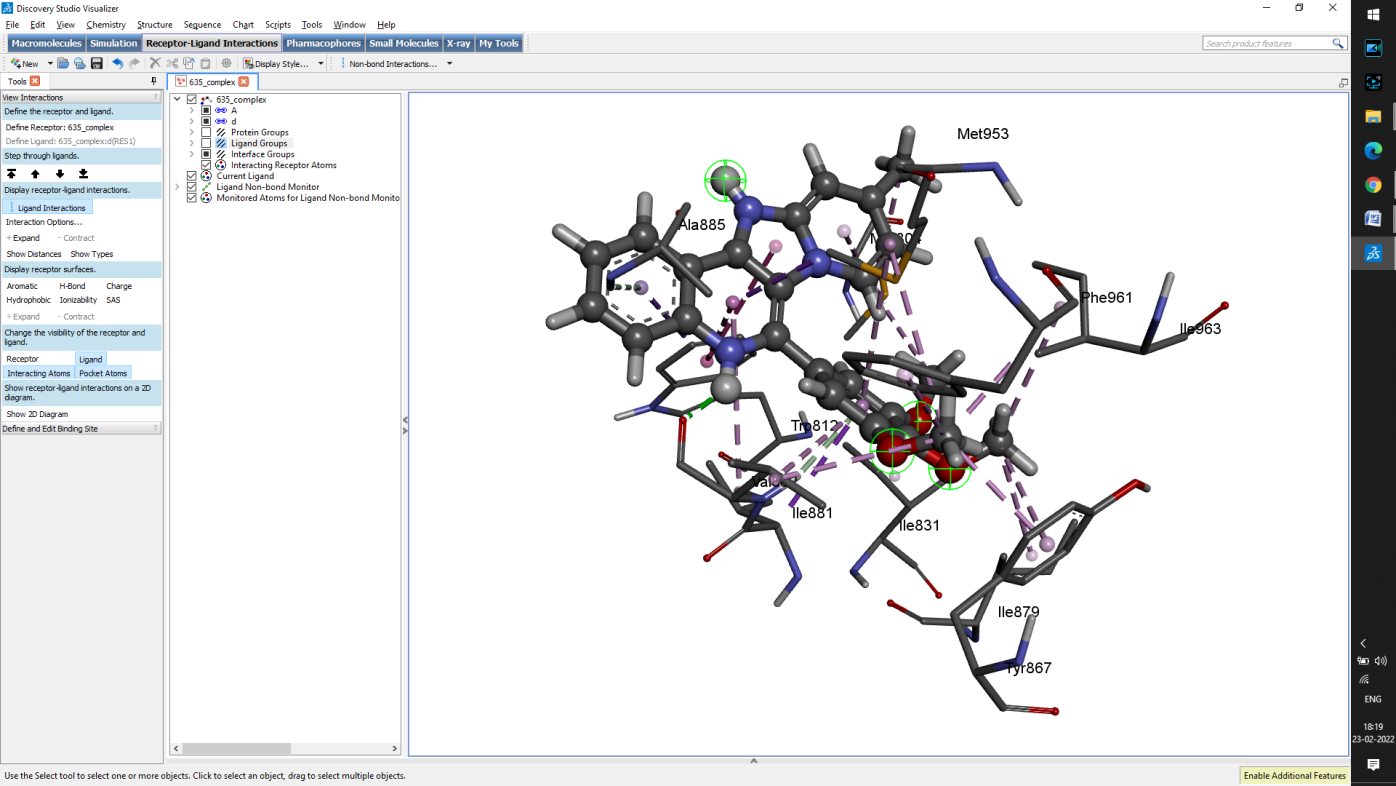 | 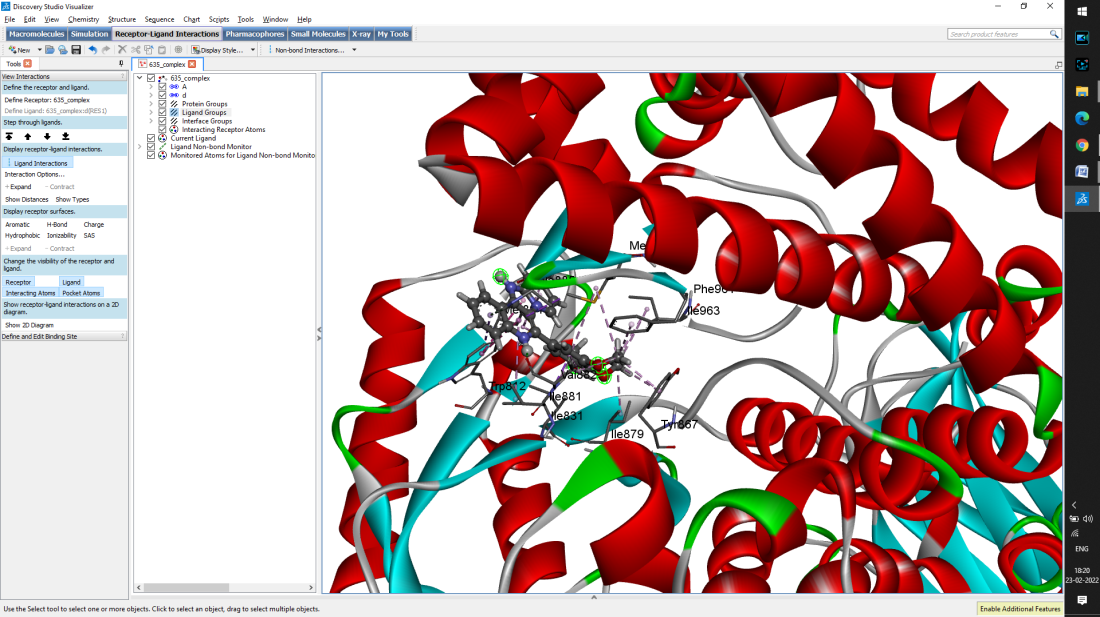 |
| **1o** | 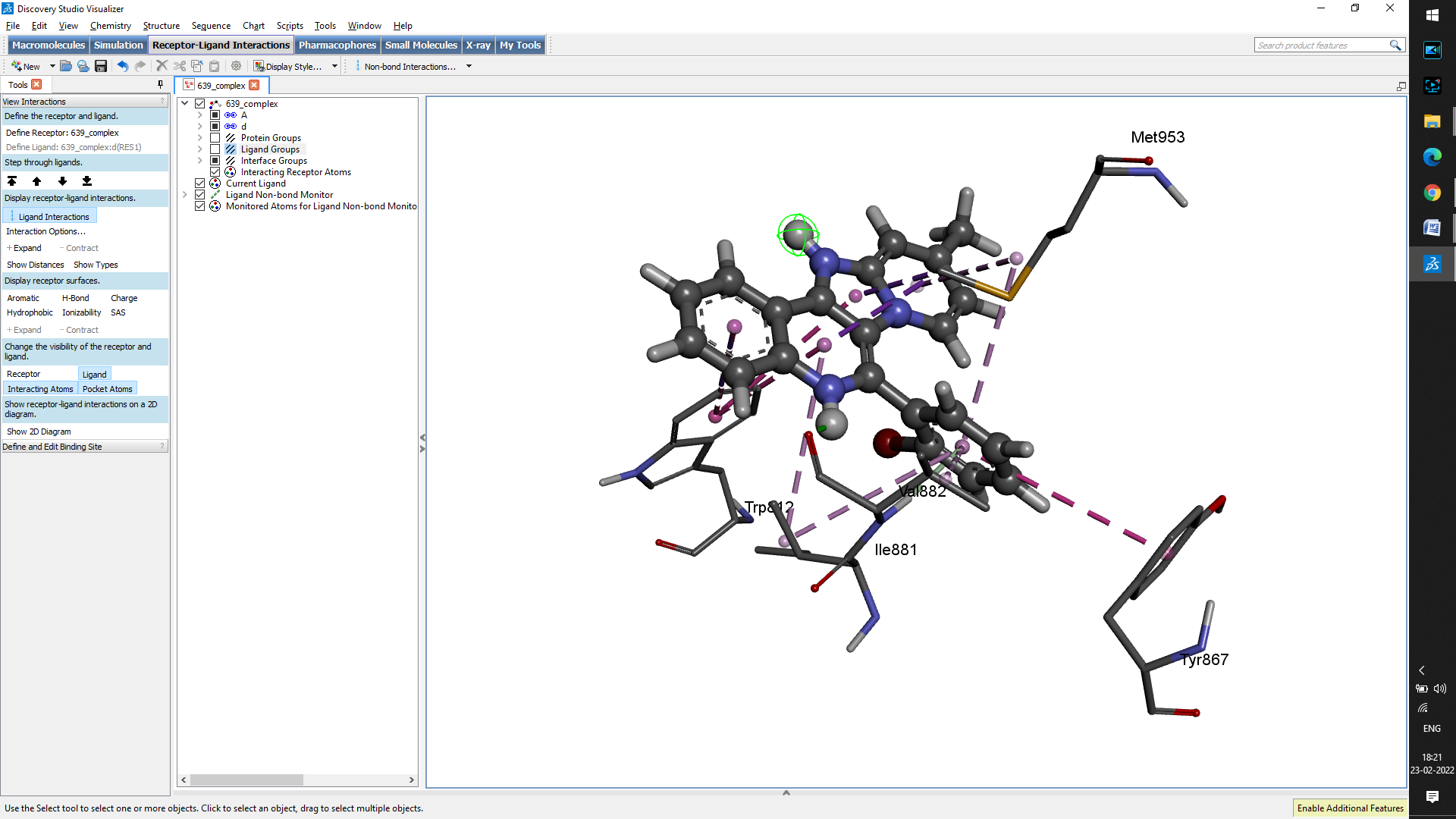 | 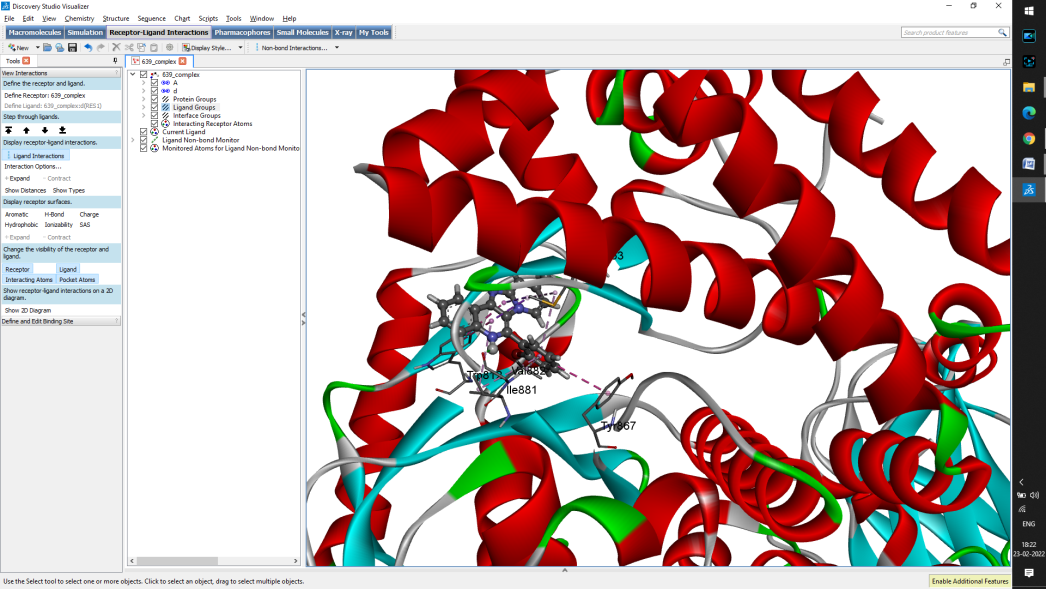 |
| **1p** | 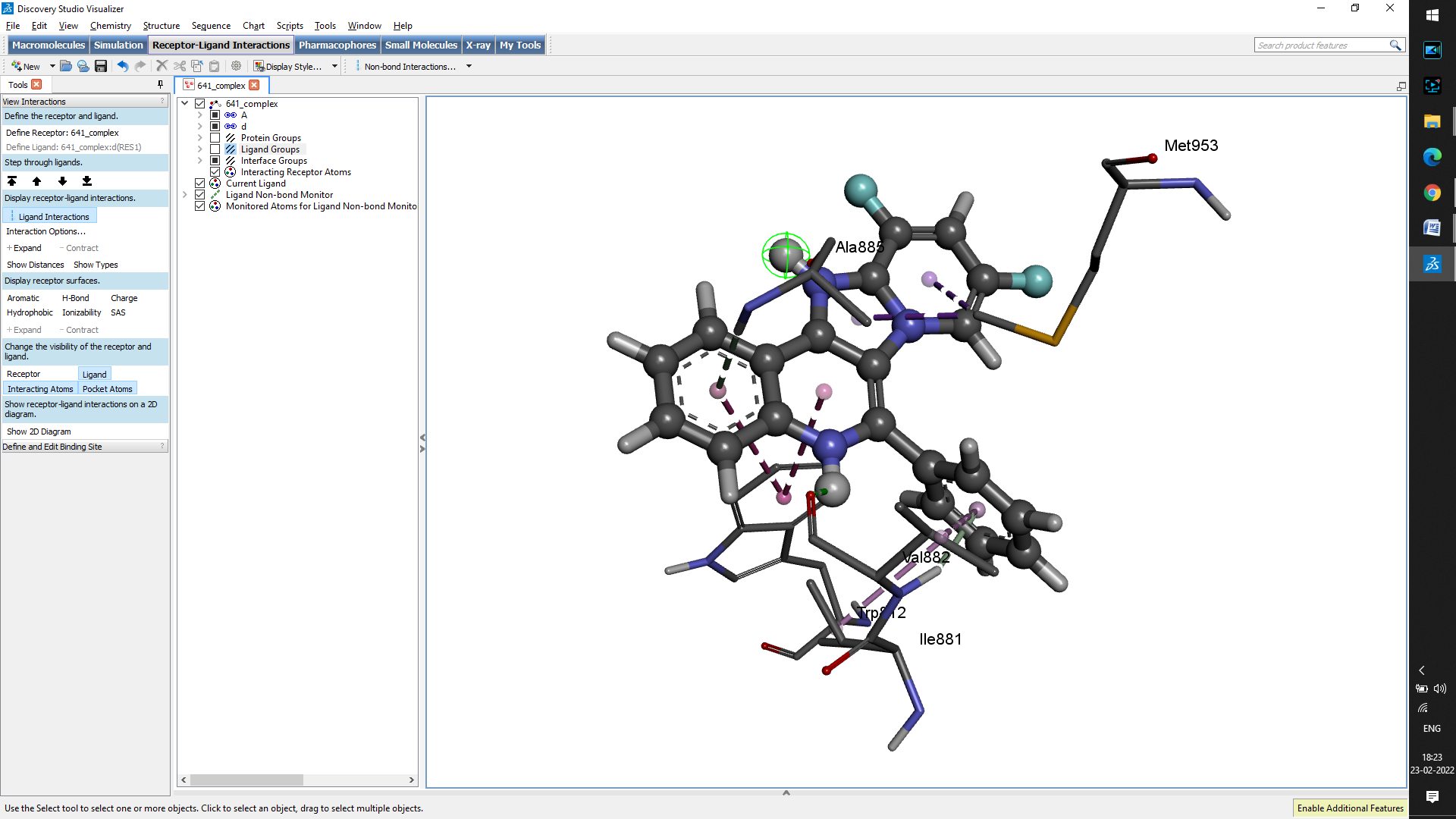 | 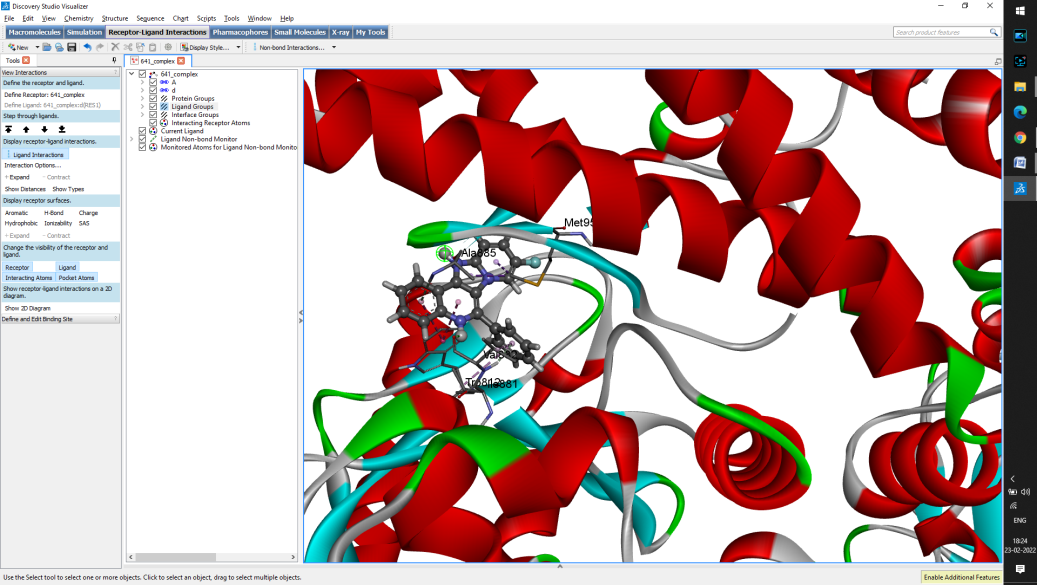 |

**Figure S1**. Docked conformation of various pyrido fused imidazo[4,5-*c*]quinolines inhibitors with PI3K.

Geometry optimized coordinates of the proposed PI3K inhibitors

**1a**

C -0.08627500 4.58766700 -0.19537700

C -1.23735100 3.76024300 -0.30387500

C -1.12055900 2.40282400 -0.24078700

N 0.11784000 1.83582500 -0.06346800

C 1.28965300 2.62351500 0.00633900

C 1.15574100 4.03046500 -0.04579100

C 0.55024700 0.50780300 0.00872900

C 1.95193700 0.59684100 0.08370200

N 2.38821600 1.88559300 0.09792500

C -0.10752500 -0.75456100 -0.02435000

N 0.60663800 -1.86057400 -0.06145500

C 1.97846300 -1.81303600 -0.01510100

C 2.72272000 -0.60158600 0.08792600

C 2.68710500 -3.03796200 -0.05041400

C 4.06364900 -3.05299000 0.01573300

C 4.79093200 -1.84816700 0.12082300

C 4.13044800 -0.63796300 0.15619200

C -1.58807400 -0.92266100 0.00162000

C -2.36677300 -0.36381900 1.02016300

C -3.74306800 -0.57887800 1.06270700

C -4.38437500 -1.35079900 0.08927800

C -3.59813600 -1.91008300 -0.92576700

C -2.22264100 -1.71071900 -0.96559400

C -5.87117100 -1.60394200 0.14558800

H 2.05682700 4.62501000 0.02410400

H -0.19684400 5.66438300 -0.24151900

H -2.21874900 4.19345400 -0.44291200

H -1.95639700 1.72655800 -0.32524900

H -1.89407600 0.22346500 1.80062200

H -4.32413700 -0.14622600 1.87091700

H -6.32289700 -1.54977400 -0.84836900

H -6.37579200 -0.87910100 0.78783300

H -6.08308100 -2.60191700 0.54440700

H -4.06977500 -2.51588400 -1.69329100

H -1.62596500 -2.16820100 -1.74565600

H 2.11199500 -3.95308800 -0.12566000

H 4.59477300 -3.99804400 -0.01101300

H 5.87347800 -1.87702200 0.17265800

H 4.67209200 0.29732300 0.23165200

**1b**

C 1.28002000 4.26456800 -0.51820100

C 2.16815300 3.16483300 -0.67109100

C 1.70425500 1.88561400 -0.57384500

N 0.37346300 1.66546200 -0.32636900

C -0.54658200 2.73076100 -0.19840000

C -0.05380300 4.05327000 -0.28771700

C -0.37856800 0.49837900 -0.19979300

C -1.70070900 0.94411500 -0.02947600

N -1.78950600 2.30256300 -0.01795800

C -0.06447600 -0.88669400 -0.24042500

N -1.03157600 -1.77795900 -0.17524000

C -2.33901200 -1.38189200 -0.02660800

C -2.74560900 -0.01901000 0.07326800

C -3.33210400 -2.38803200 0.04407300

C -4.65916600 -2.05308400 0.20625800

C -5.05122900 -0.70107100 0.30560200

C -4.10816900 0.30313400 0.24002400

C 1.31971500 -1.42838800 -0.37837600

C 1.62895600 -2.26915100 -1.44791700

C 2.89611300 -2.83328300 -1.58516100

C 3.86724100 -2.56766800 -0.62614100

C 3.57938100 -1.74805200 0.46596900

C 2.30902300 -1.17991900 0.59418900

O 1.93554700 -0.37722700 1.63327200

C 2.85008000 -0.15940400 2.70101100

H -0.76161300 4.86408000 -0.17835900

H 1.66444100 5.27471700 -0.59342700

H 3.21876100 3.32643100 -0.87193900

H 2.32681600 1.01335000 -0.69600500

H 0.85237500 -2.48212500 -2.17282500

H 3.11677800 -3.47721800 -2.42799400

H 4.85664600 -3.00230100 -0.71466500

H 4.34110100 -1.56851800 1.21253600

H 3.75035300 0.36194400 2.35830100

H 2.32235700 0.46675500 3.41814600

H 3.13189800 -1.10288000 3.17937800

H -3.01187000 -3.42047400 -0.02988500

H -5.41007300 -2.83363200 0.25981600

H -6.09905900 -0.45358900 0.43331300

H -4.39100100 1.34663900 0.31095000

**1c**

C -1.62008100 4.59684700 -0.97155900

C -0.35321900 3.95225100 -0.95143300

C -0.25753400 2.62558600 -0.64962200

N -1.39411800 1.91182000 -0.35792300

C -2.67462300 2.50824900 -0.40889100

C -2.76143000 3.88794300 -0.70707400

C -1.61579500 0.56489000 -0.05093000

C -3.01582400 0.44826800 0.02673500

N -3.64663500 1.63637800 -0.17569500

C -0.77279500 -0.57007400 0.12284300

N -1.31117100 -1.76230800 0.28029100

C -2.67380600 -1.91814400 0.33839700

C -3.59498600 -0.83469600 0.24645600

C -3.18702100 -3.22483400 0.52143100

C -4.54576800 -3.43783800 0.60987000

C -5.44937000 -2.35747600 0.52047500

C -4.98139300 -1.07269500 0.34047400

C 0.71659200 -0.51154900 0.16104200

C 1.38134700 0.35720200 1.01849900

C 2.78034900 0.36061500 1.09572300

C 3.52931500 -0.52149300 0.30185900

C 2.84262100 -1.41335300 -0.55502900

C 1.45372200 -1.40292200 -0.62818300

O 3.63358500 -2.27987600 -1.25187000

C 3.00756000 -3.29485400 -2.03048200

O 4.88757200 -0.60153200 0.47198800

C 5.71592500 -0.29722300 -0.65841900

O 3.30608700 1.20147100 2.03418200

C 4.55285400 1.86656200 1.81184200

H -3.74509500 4.33770400 -0.72735700

H -1.67823100 5.65281500 -1.20659600

H 0.55058900 4.50237600 -1.17650200

H 0.67424100 2.08351900 -0.62016400

H 0.84258300 1.02743300 1.67732600

H 5.39860400 1.20672100 2.00104000

H 4.56405400 2.69974200 2.51479300

H 4.61130600 2.25764400 0.79068800

H 5.56643200 -1.01649800 -1.46351300

H 6.74300100 -0.35304200 -0.29839400

H 5.51243400 0.71621100 -1.02286400

H 2.36850800 -3.93079300 -1.41045700

H 3.81952500 -3.88937800 -2.44600100

H 2.41638400 -2.86581500 -2.84609300

H 0.92146800 -2.10063600 -1.25753300

H -2.47848900 -4.04117700 0.59568700

H -4.92612500 -4.44328100 0.75263100

H -6.51536700 -2.54122900 0.59327600

H -5.66013300 -0.23161600 0.26478700

**1d**

C -3.80556909 -2.72760623 -0.15568986

C -3.87412053 -1.30068026 -0.12051471

C -2.73757106 -0.53660378 -0.07282212

N -1.57420046 -1.16904730 -0.06054719

C -1.45856951 -2.59261071 -0.09447772

C -2.60064787 -3.37646188 -0.14281636

C -0.22305961 -0.69934259 -0.01518491

C 0.56192762 -1.81212866 -0.02462841

N -0.18762249 -2.95906539 -0.07268965

C 0.38285617 0.69244219 0.03566739

N 1.68716732 0.76675101 0.06966046

C 2.48012141 -0.35107717 0.06028956

C 1.94309529 -1.66323182 0.01318795

C 3.87219874 -0.20072356 0.09841231

C 4.68033849 -1.29330062 0.09006247

C 4.13736900 -2.58933597 0.04322867

C 2.79419656 -2.77183608 0.00548004

C -0.49773523 1.95578642 0.04664800

C -1.00340836 2.44993046 1.25849374

C -1.88671049 3.53788800 1.26561625

C -2.29290107 4.11835730 0.05836853

C -1.82717654 3.60043095 -1.15581243

C -0.94390845 2.51243715 -1.16145917

C -0.50292540 1.97873122 -2.37990661

N -0.14212515 1.54205963 -3.37681797

H -2.54267410 -4.44456973 -0.16898262

H -4.71034779 -3.29761341 -0.19266701

H -4.83010947 -0.82019946 -0.13161582

H -2.78828993 0.53186864 -0.04647209

H -0.71228030 1.99455732 2.18195447

H -2.25166692 3.92432877 2.19425521

H -2.95989628 4.95501149 0.06357948

H -2.14657789 4.03472954 -2.08007838

H 4.30128458 0.77881697 0.13426910

H 5.74229488 -1.16569383 0.11947597

H 4.78890593 -3.43807905 0.03732659

H 2.38744106 -3.76086886 -0.03007522

**1e**

C -1.39418200 4.71221800 0.00000000

C -2.37791200 3.69273600 0.00000000

C -2.02675700 2.37546600 0.00000000

N -0.70684900 1.97439500 0.00000000

C 0.28484800 2.98697600 0.00000000

C -0.07749700 4.35394600 0.00000000

C 0.00000000 0.73344600 0.00000000

C 1.35854900 1.15635400 0.00000000

N 1.50890500 2.49859800 0.00000000

C -0.27944900 -0.70445000 0.00000000

N 0.78802100 -1.50458700 0.00000000

C 2.09375400 -1.11544400 0.00000000

C 2.45140800 0.24562200 0.00000000

C 3.11332400 -2.09748700 0.00000000

C 4.43604400 -1.71103000 0.00000000

C 4.78747700 -0.34369900 0.00000000

C 3.80773700 0.62721900 0.00000000

C -1.55951000 -1.47903600 0.00000000

C -2.83630500 -0.89399700 0.00000000

C -4.02451500 -1.60390800 0.00000000

C -3.98298300 -2.99932400 0.00000000

C -2.75911600 -3.63749700 0.00000000

C -1.55043000 -2.91983500 0.00000000

O -0.44003400 -3.65936200 0.00000000

H 0.73114500 5.07242700 0.00000000

H -1.68792100 5.75455900 0.00000000

H -3.43208000 3.93620600 0.00000000

H -2.78969800 1.62860300 0.00000000

H -2.91327400 0.16910200 0.00000000

H -4.96978300 -1.07478400 0.00000000

H -4.89872400 -3.57996500 0.00000000

H -2.68321800 -4.71810600 0.00000000

H 0.30770100 -2.97517700 0.00000000

H 2.83482800 -3.14480600 0.00000000

H 5.21612100 -2.46391900 0.00000000

H 5.83341900 -0.05927700 0.00000000

H 4.05431300 1.68153100 0.00000000

**1f**

C 0.48449500 4.25658100 -0.20104400

C 1.64432700 3.43985600 -0.29017000

C 1.54378600 2.08299400 -0.22427200

N 0.30376000 1.51259800 -0.06291300

C -0.86970300 2.29197700 -0.01146300

C -0.76836300 3.71060100 -0.06664900

C -0.12103000 0.18174800 0.01005100

C -1.52352400 0.26082700 0.06897200

N -1.96641700 1.54758300 0.07096600

C 0.54428200 -1.07631600 -0.00909700

N -0.16107700 -2.18800600 -0.04384900

C -1.53332900 -2.14982000 -0.00977500

C -2.28644600 -0.94233000 0.07453800

C -2.23314800 -3.38006800 -0.04091100

C -3.61001100 -3.40409600 0.01116800

C -4.34640000 -2.20316000 0.09798700

C -3.69470100 -0.98821400 0.12864100

C 2.02684400 -1.23656400 0.02865400

C 2.78769600 -0.68051800 1.06439200

C 4.16508300 -0.88576200 1.11981100

C 4.79937900 -1.64190000 0.13516400

C 4.04730100 -2.20441500 -0.89585200

C 2.66917200 -2.01130700 -0.94398400

C -2.02826600 4.52359100 0.01026500

H 0.59309300 5.33433200 -0.25040600

H 2.62186800 3.88580500 -0.41668600

H 2.38502700 1.41250600 -0.29417600

H 2.29758900 -0.10684500 1.84399200

H 4.73983700 -0.46311200 1.93638500

H 5.87132400 -1.79921700 0.17609500

H 4.53396100 -2.80036000 -1.65987300

H 2.07725700 -2.46437800 -1.73006100

H -1.65091700 -4.29173200 -0.10225200

H -4.13447300 -4.35293100 -0.01241400

H -5.42921200 -2.23903600 0.13921200

H -4.24373600 -0.05623100 0.19037500

H -2.70899100 4.26116500 -0.80424500

H -1.80582700 5.59046200 -0.04518100

H -2.56530900 4.32211600 0.94125900

**1g**

C 0.41533700 4.21352400 -0.49538800

C 1.52317800 3.33834100 -0.66176000

C 1.35844200 1.98885000 -0.57175100

N 0.10685700 1.48622400 -0.31816200

C -1.01843200 2.32324000 -0.17923200

C -0.84983200 3.73396200 -0.25829800

C -0.37222500 0.18190100 -0.19745400

C -1.75815100 0.32651500 -0.01948900

N -2.13905900 1.63379100 0.00296800

C 0.23640600 -1.10102800 -0.24826800

N -0.51289400 -2.18259300 -0.18976600

C -1.87451000 -2.08227300 -0.03543600

C -2.56765500 -0.84152200 0.07844700

C -2.62452700 -3.28122400 0.02839000

C -3.99173300 -3.24507700 0.19843400

C -4.66820800 -2.01175400 0.31291000

C -3.96687900 -0.82582700 0.25362200

C 1.70575000 -1.32648500 -0.38492200

C 2.19527500 -2.07209900 -1.45747000

C 3.55600800 -2.34283100 -1.59210300

C 4.44191100 -1.87558900 -0.62757800

C 3.97780400 -1.14572200 0.46756200

C 2.61319600 -0.87214000 0.59340800

C -2.05640300 4.61213700 -0.09215800

O 2.06848600 -0.17886300 1.63527700

C 2.90963200 0.23201700 2.70616900

H 0.57367400 5.28399600 -0.56618200

H 2.50912000 3.73350800 -0.86750300

H 2.15452900 1.27360600 -0.70304100

H 1.48660200 -2.44561000 -2.18700000

H 3.91578400 -2.91746000 -2.43714600

H 5.50288800 -2.08186600 -0.71412600

H 4.67930200 -0.80793300 1.21832900

H 2.25487400 0.72279100 3.42403000

H 3.39252600 -0.62740300 3.18239800

H 3.67284500 0.94129200 2.36820100

H -2.08742800 -4.21836500 -0.05693600

H -4.55432300 -4.17090200 0.24681600

H -5.74406000 -1.99960400 0.44703300

H -4.47007700 0.13023100 0.33583600

H -2.81281500 4.37864000 -0.84651100

H -1.78494000 5.66564000 -0.17852900

H -2.52700600 4.44688700 0.88103100

**1h**

C -1.38477100 4.24046100 -0.22900400

C -0.03873700 3.79022900 -0.30373600

C 0.25382300 2.46206200 -0.22421200

N -0.77110300 1.56068100 -0.06277300

C -2.11932900 1.97083700 -0.02953500

C -2.42898700 3.35845400 -0.09706400

C -0.79665200 0.16414500 0.02131300

C -2.16341600 -0.16180400 0.06383500

N -2.95721000 0.94371300 0.04957000

C 0.20387200 -0.84993600 0.02219200

N -0.15505800 -2.11757200 -0.01718500

C -1.48106600 -2.47366700 -0.00635000

C -2.54977000 -1.53301100 0.06499600

C -1.79953500 -3.85275400 -0.04428200

C -3.11254700 -4.27015500 -0.01127200

C -4.16310200 -3.33065800 0.06268400

C -3.88651200 -1.98006800 0.09973700

C 1.66561200 -0.57540100 0.08513100

C 2.22040200 0.21420400 1.10422000

C 3.58988400 0.41338400 1.19151500

C 4.44594400 -0.17105700 0.24940400

C 3.91080100 -0.96844600 -0.76656600

C 2.53276200 -1.17071500 -0.83213200

C -3.86997000 3.77559300 -0.03497300

O 5.77383200 0.09174800 0.40974400

C 6.69872500 -0.49137500 -0.49980200

H -1.58984300 5.30360400 -0.28769900

H 0.77096200 4.49670200 -0.42927400

H 1.25250200 2.06071300 -0.28397400

H 1.57472100 0.65690700 1.85525200

H 4.02096300 1.00786400 1.98802100

H 6.66250900 -1.58514200 -0.45898900

H 7.68252800 -0.15070000 -0.18168100

H 6.51373200 -0.15736100 -1.52635400

H 4.54923400 -1.43912100 -1.50211100

H 2.12179400 -1.80726900 -1.60644200

H -0.98002100 -4.55974100 -0.09447000

H -3.34285400 -5.32948300 -0.03939900

H -5.19083400 -3.67498400 0.08927300

H -4.68017300 -1.24426500 0.15092300

H -4.44025000 3.32236500 -0.85051200

H -3.96310300 4.86096600 -0.09948100

H -4.33352600 3.43528700 0.89508600

**1i**

C -2.01958900 4.21339900 -0.82111400

C -0.68060200 3.73698100 -0.83544300

C -0.40576200 2.43026200 -0.56624800

N -1.44163700 1.57556100 -0.27424200

C -2.78348400 2.00637700 -0.29657700

C -3.07472600 3.37465700 -0.55924400

C -1.48678100 0.20420500 0.00358700

C -2.85888400 -0.09131300 0.09238200

N -3.63605100 1.01317900 -0.07378100

C -0.50506200 -0.81912000 0.14164400

N -0.88711100 -2.07369000 0.27201600

C -2.21795700 -2.40246300 0.33605000

C -3.26945900 -1.44207300 0.28237600

C -2.55997300 -3.76831600 0.48542100

C -3.87974700 -4.15427400 0.57690200

C -4.91355300 -3.19472600 0.52577900

C -4.61361000 -1.85654600 0.38029000

C 0.96552500 -0.57538900 0.16948200

C 1.52419600 0.36301800 1.02924500

C 2.91192100 0.54462000 1.09113500

C 3.75773200 -0.22823200 0.28114700

C 3.18078900 -1.19444100 -0.57624600

C 1.80122200 -1.36029500 -0.63482800

C -4.50956400 3.81671000 -0.54937800

O 3.33830000 1.44030300 2.02978000

C 4.48187600 2.26571700 1.79164100

O 5.11735700 -0.13264300 0.43295400

C 5.88090300 0.28630600 -0.70638900

O 4.06769500 -1.94716800 -1.29011200

C 3.56521200 -3.02365100 -2.07571200

H -2.21036000 5.26007300 -1.03077900

H 0.13822900 4.40670300 -1.06236800

H 0.58676100 2.00999600 -0.56380400

H 0.91155200 0.95490300 1.69822800

H 1.35419800 -2.11445200 -1.26533800

H -1.75301800 -4.48993900 0.53088400

H -4.12850400 -5.20339300 0.69281300

H -5.94694100 -3.51400100 0.60103600

H -5.39401000 -1.10638600 0.33445700

H -5.09237300 3.25950600 -1.28800400

H -4.58895500 4.88296600 -0.76780000

H -4.97017400 3.61978600 0.42262500

H 5.41072100 1.72462900 1.96783800

H 4.39202800 3.09310800 2.49583900

H 4.47333400 2.66088900 0.77048700

H 5.80376600 -0.43429200 -1.52041100

H 6.91397900 0.35053000 -0.36548900

H 5.54883500 1.27262100 -1.05032400

H 3.01849600 -3.74219000 -1.45767000

H 4.44044900 -3.50638200 -2.50742600

H 2.91501700 -2.66243000 -2.87914700

**1j**

C -1.49982300 4.26056700 -0.25374700

C -0.16724800 3.78219000 -0.37845600

C 0.10011800 2.44882200 -0.30630100

N -0.93665400 1.56901600 -0.10243200

C -2.27380800 2.00757300 -0.02123800

C -2.55693500 3.40132400 -0.08092100

C -0.98961100 0.17406300 -0.01340000

C -2.36174700 -0.12334800 0.07986200

N -3.12993400 0.99842300 0.09127700

C -0.02036500 -0.86608400 -0.04291500

N -0.40474400 -2.12578700 -0.06240200

C -1.73579100 -2.45291500 0.00404800

C -2.77808300 -1.48531300 0.10557400

C -2.08524300 -3.82482300 -0.01017400

C -3.40536000 -4.21005400 0.07273300

C -4.43013000 -3.24470900 0.17566400

C -4.12262700 -1.90063800 0.19178900

C 1.45406200 -0.64619300 -0.01990200

C 2.05348400 0.13765000 0.97558600

C 3.43431800 0.28825400 1.02766600

C 4.21096400 -0.34709700 0.06451800

C 3.64675900 -1.14123900 -0.92952100

C 2.26664800 -1.29319900 -0.96015800

C -3.98533300 3.84871500 0.03376100

N 5.67915200 -0.17968300 0.10442300

O 6.14629100 0.53624900 0.98222200

O 6.34160900 -0.76374200 -0.74393100

H -1.68408400 5.32760100 -0.30802300

H 0.65115400 4.47140100 -0.53838700

H 1.08791500 2.02964300 -0.40698100

H 1.43957300 0.61124300 1.73303700

H 3.91187000 0.87733900 1.79836700

H 4.28593900 -1.62586700 -1.65461400

H 1.80486200 -1.92188600 -1.71064200

H -1.28488000 -4.55128300 -0.08339400

H -3.66187100 -5.26345600 0.06169900

H -5.46377100 -3.56490800 0.24140000

H -4.89695600 -1.14655700 0.26522900

H -4.59580300 3.40194400 -0.75566500

H -4.05856200 4.93515600 -0.03507500

H -4.41883100 3.52452400 0.98384000

**1k**

C 3.44583200 -2.46213700 -0.13352400

C 3.54918800 -1.03987200 -0.23741700

C 2.44613500 -0.24271300 -0.18781400

N 1.20524300 -0.80726400 -0.02767300

C 1.04319100 -2.20696500 0.03608900

C 2.19930500 -3.02116100 -0.00176300

C -0.08655100 -0.27111300 0.02712000

C -0.92994600 -1.39472100 0.08835800

N -0.23295200 -2.56406200 0.10902000

C -0.61767200 1.04748700 -0.00946700

N -1.92211700 1.22626800 -0.05882700

C -2.77741400 0.15298900 -0.02336000

C -2.34112400 -1.20081500 0.07784300

C -4.16888100 0.41075800 -0.07056300

C -5.07556300 -0.62552200 -0.01812700

C -4.63349400 -1.96211200 0.08433900

C -3.28517300 -2.24716000 0.13105700

C 0.21838800 2.28207700 0.02574300

C 1.13320900 2.50567300 1.06224000

C 1.86936800 3.68795700 1.11455800

C 1.70643500 4.65716000 0.12580100

C 0.79186000 4.44491000 -0.90556800

C 0.04617800 3.26984400 -0.95070000

C 4.69426700 -3.30210100 -0.18024500

H 2.05432500 -4.09199400 0.06405900

H 4.51792400 -0.57266700 -0.36383800

H 2.48491000 0.83195000 -0.27112000

H 1.25049000 1.76412600 1.84556300

H 2.56182100 3.85506800 1.93211800

H 2.28115500 5.57570000 0.16401500

H 0.65417700 5.19898400 -1.67250600

H -0.68112300 3.10957100 -1.73742200

H -4.48956900 1.44307800 -0.14377900

H -6.13870700 -0.41480400 -0.05358400

H -5.35956900 -2.76624700 0.12500100

H -2.92734100 -3.26717200 0.20444400

H 4.46219000 -4.36554600 -0.10801900

H 5.36929700 -3.04417600 0.64208600

H 5.24358000 -3.13524800 -1.11226100

**1l**

C 0.29049600 4.30813000 -0.13383800

C 1.39185000 3.40431800 -0.25412800

C 1.21120900 2.05518500 -0.20674800

N -0.05019200 1.54317800 -0.03145900

C -1.17598800 2.38923000 0.04922400

C -0.97117900 3.78833900 0.01330800

C -0.55034800 0.23683300 0.02370100

C -1.94486800 0.39891900 0.10119800

N -2.31266400 1.70956100 0.13276700

C 0.04025200 -1.05644400 -0.02523700

N -0.73061600 -2.12433200 -0.07588500

C -2.09727600 -2.00508600 -0.02822100

C -2.77693300 -0.75720100 0.09071900

C -2.87013900 -3.19043700 -0.07947700

C -4.24537700 -3.13395000 -0.01355600

C -4.90833300 -1.89370300 0.10740400

C -4.18504400 -0.72081000 0.15841900

C 1.50983300 -1.30198800 -0.00089000

C 2.10142400 -2.12224800 -0.96831400

C 3.46486800 -2.39335400 -0.92999900

C 4.28053600 -1.87605800 0.08374300

C 3.68151600 -1.07093100 1.05749000

C 2.31845000 -0.78448700 1.01650800

C 0.53921600 5.79214800 -0.18126000

C 5.75151700 -2.20889400 0.14154600

H -1.84671100 4.42013100 0.09277900

H 2.39586800 3.78586700 -0.39231900

H 2.01588600 1.34339500 -0.30308700

H 1.48072400 -2.54812500 -1.74750800

H 3.90318300 -3.02283500 -1.69821600

H 4.28524900 -0.66837300 1.86477200

H 1.87827500 -0.17328400 1.79762100

H -2.34399400 -4.13355400 -0.16688800

H -4.82550200 -4.04932700 -0.05282900

H -5.99095800 -1.86609800 0.15883000

H -4.67679700 0.24065400 0.24603400

H -0.39154200 6.35492700 -0.09756600

H 1.02691100 6.07717500 -1.11887300

H 1.20032000 6.10395800 0.63371200

H 6.30630600 -1.46986900 0.72371000

H 6.19005800 -2.25299500 -0.85852700

H 5.91292400 -3.18556900 0.61085000

**1m**

C -1.16121700 4.28214300 -0.16024300

C 0.14364700 3.70288200 -0.23723000

C 0.32668400 2.35481400 -0.17363800

N -0.75787500 1.52690800 -0.02388500

C -2.06989100 2.04368100 0.01307300

C -2.24338700 3.44666400 -0.03933000

C -0.89420100 0.13503500 0.04010600

C -2.28320000 -0.07914400 0.07795300

N -2.98695100 1.08669600 0.07798300

C 0.02155400 -0.95408400 0.02472800

N -0.43653400 -2.18910600 -0.03045600

C -1.78663300 -2.43752300 -0.02108300

C -2.77722300 -1.41522900 0.06256600

C -2.21460000 -3.78616900 -0.07488400

C -3.55667700 -4.09760200 -0.04459700

C -4.52905600 -3.07813000 0.04170200

C -4.14559900 -1.75456200 0.09378400

C 1.50041200 -0.79571200 0.08635400

C 2.11747600 -0.06430400 1.11293000

C 3.49861900 0.02685500 1.19813400

C 4.30433300 -0.61131600 0.24662200

C 3.70623300 -1.35315600 -0.77637500

C 2.31652000 -1.44626700 -0.84024100

C -1.31335900 5.77858100 -0.22256300

O 5.64922700 -0.45562800 0.40584300

C 6.52326900 -1.09947400 -0.51299600

H -3.25730000 3.82341300 0.00675400

H 1.01400900 4.33652600 -0.35421500

H 1.29437000 1.88289300 -0.23766300

H 1.50972800 0.41691800 1.87199700

H -2.36186900 6.07535800 -0.17073500

H -0.89174200 6.17643200 -1.15111700

H -0.78198800 6.26015300 0.60460800

H 6.39971600 -2.18727800 -0.48589800

H 7.53158700 -0.84275300 -0.19281400

H 6.36435300 -0.73881600 -1.53491600

H -1.45440200 -4.55590000 -0.13456000

H -3.87093800 -5.13475900 -0.08465100

H -5.58096900 -3.33968200 0.06587200

H -4.87742100 -0.95793000 0.15476300

H 1.85547200 -2.04053100 -1.61994400

H 4.30423100 -1.86435200 -1.51882300

H 3.97665100 0.57594600 2.00046100

**1n**

C -1.94563200 4.26932900 -0.66458300

C -0.62704800 3.71717000 -0.68113800

C -0.40892200 2.39250200 -0.45222800

N -1.47060800 1.56224500 -0.19291000

C -2.79475900 2.04757800 -0.20829000

C -3.00485400 3.42870900 -0.43064400

C -1.57112100 0.18468300 0.03896100

C -2.95505700 -0.05598500 0.11290900

N -3.68739200 1.08372600 -0.02130700

C -0.63275000 -0.88027900 0.14379900

N -1.06459600 -2.12263800 0.23426800

C -2.40774900 -2.39816000 0.28585300

C -3.41965900 -1.39450500 0.25875200

C -2.80606400 -3.75304000 0.39067100

C -4.14080700 -4.08744700 0.46453700

C -5.13484000 -3.08553200 0.43940800

C -4.78017700 -1.75685800 0.33781500

C 0.84643600 -0.69524700 0.17491100

C 1.44397500 0.18988700 1.06443700

C 2.83810700 0.31587800 1.12637400

C 3.65050100 -0.45895800 0.28459600

C 3.03346500 -1.37074400 -0.60385100

C 1.64844800 -1.48200600 -0.66125900

C -2.13689000 5.74235300 -0.90943400

O 3.88849800 -2.13044300 -1.34846500

C 3.34414700 -3.16658800 -2.16004800

O 5.01363100 -0.42264600 0.43231600

C 5.78759000 0.01158100 -0.69406400

O 3.29971300 1.15995900 2.09563800

C 4.48450700 1.93572700 1.89559900

H -4.02785400 3.78285600 -0.42065800

H 0.22613500 4.35335600 -0.88137100

H 0.56970900 1.93943300 -0.45822100

H 0.85704200 0.77917000 1.75839800

H 5.38417000 1.34205500 2.05261800

H 4.43177200 2.73681100 2.63316800

H 4.50183400 2.37264200 0.89168500

H -3.19217500 6.01754600 -0.88163900

H -1.73431500 6.03231900 -1.88522100

H -1.61056100 6.33510600 -0.15441800

H 5.68004900 -0.67330300 -1.53507900

H 6.82382400 0.02383600 -0.35687100

H 5.49139000 1.02231300 -0.99763300

H 2.77483600 -3.88152000 -1.55849500

H 4.19963100 -3.66837500 -2.60929200

H 2.70378800 -2.76123300 -2.95025400

H -2.02948600 -4.50819700 0.41611300

H -4.43252400 -5.12874000 0.54603000

H -6.18068200 -3.36486200 0.50002000

H -5.52890000 -0.97417400 0.31229900

H 1.17067100 -2.19563200 -1.31594400

**1o**

C 2.14338200 3.74293700 -0.29234300

C 2.69971100 2.44887200 -0.53700500

C 1.91663200 1.33510000 -0.56422800

N 0.56631200 1.45124600 -0.35469700

C -0.03710200 2.70380000 -0.12019100

C 0.78965000 3.85044300 -0.08912000

C -0.47201500 0.51929400 -0.33293200

C -1.62934100 1.28166300 -0.09813200

N -1.35284700 2.60779100 0.03502300

C -0.54026500 -0.88505600 -0.50920900

N -1.70243100 -1.50032700 -0.49123900

C -2.85766400 -0.79066800 -0.26425400

C -2.88940300 0.61911600 -0.04795800

C -4.07957100 -1.50461600 -0.24028900

C -5.26954200 -0.84872700 -0.01114200

C -5.29084600 0.54633000 0.20264300

C -4.11822600 1.27129300 0.18407900

C 0.65924500 -1.74175700 -0.76837800

C 0.84172200 -2.27837200 -2.04992500

C 1.92705500 -3.09681600 -2.34449300

C 2.85262000 -3.40410000 -1.34907400

C 2.68612000 -2.89830800 -0.06259900

C 1.59610800 -2.07578500 0.21580000

Br 1.39043400 -1.43648200 2.01575200

C 3.04843700 4.94574600 -0.26614500

H 0.31143600 4.80373700 0.09658900

H 3.76369300 2.33704700 -0.70399600

H 2.29828400 0.34249000 -0.74737800

H 0.10848700 -2.04572200 -2.81319900

H 2.04617700 -3.49710600 -3.34450000

H 3.70039200 -4.04409800 -1.56529000

H 3.38824600 -3.14663800 0.72280000

H -4.04301200 -2.57509300 -0.40325900

H -6.19881500 -1.40728800 0.00712300

H -6.23529100 1.04747900 0.38251000

H -4.11568300 2.34272700 0.34479300

H 2.48805200 5.86207200 -0.07547200

H 3.57467600 5.05984800 -1.21920500

H 3.81070000 4.84412100 0.51292400

**1p**

C -3.58911600 -2.41108400 0.00000000

C -3.67366400 -1.00333100 0.00000000

C -2.59220700 -0.18316500 0.00000000

N -1.32030700 -0.72148500 0.00000000

C -1.17290100 -2.12634800 0.00000000

C -2.33621700 -2.93915100 0.00000000

C 0.00000000 -0.19860500 0.00000000

C 0.80176600 -1.37405400 0.00000000

N 0.08038900 -2.52003200 0.00000000

C 0.66914400 1.10030100 0.00000000

N 1.98831300 1.09191600 0.00000000

C 2.76802800 -0.02113700 0.00000000

C 2.22127700 -1.32357700 0.00000000

C 4.17716300 0.12867800 0.00000000

C 4.98852700 -0.98445800 0.00000000

C 4.43030300 -2.28228700 0.00000000

C 3.06197100 -2.45454600 0.00000000

C 0.12725900 2.51564100 0.00000000

C -1.21529700 2.90803900 0.00000000

C -1.60416000 4.24781500 0.00000000

C -0.65062400 5.25638800 0.00000000

C 0.69726800 4.89783900 0.00000000

C 1.07516300 3.56311200 0.00000000

F -2.14953900 -4.26213600 0.00000000

F -4.90158500 -0.43937000 0.00000000

H -4.47491300 -3.03110600 0.00000000

H -2.73130600 0.87521400 0.00000000

H -2.00521700 2.18796200 0.00000000

H -2.66065800 4.49124100 0.00000000

H -0.94792200 6.29880100 0.00000000

H 1.46428000 5.66438300 0.00000000

H 2.12160300 3.30100500 0.00000000

H 4.58681800 1.13150200 0.00000000

H 6.06642000 -0.86711500 0.00000000

H 5.08485400 -3.14641900 0.00000000

H 2.61530100 -3.44112700 0.00000000

**Toxicity analysis using ProTox-II Prediction of toxicity, an online application.^1^**

| Target | **Ref** | **1a** | **1b** | **1c** | **1d** | **1e** | **1f** | **1g** | **1h** | **1i** | **1j** | **1k** | **1l** | **1m** | **1n** | **1o** | **1p** |
| --- | --- | --- | --- | --- | --- | --- | --- | --- | --- | --- | --- | --- | --- | --- | --- | --- | --- |
| Hepatotoxicity | 0.88 | 0.52 | 0.56 | 0.61 | 0.55 | 0.52 | 0.52 | 0.61 | 0.58 | 0.60 | 0.53 | 0.52 | 0.59 | 0.58 | 0.60 | 0.51 | 0.64 |
| Carcinogenicity | 0.52 | 0.59 | 0.51 | 0.51 | 0.60 | 0.58 | 0.59 | 0.51 | 0.56 | 0.52 | 0.84 | 0.59 | 0.58 | 0.56 | 0.52 | 0.61 | 0.64 |
| Cytotoxicity | 0.77 | 0.96 | 0.73 | 0.80 | 0.89 | 0.71 | 0.96 | 0.73 | 0.73 | 0.80 | 0.84 | 0.96 | 0.95 | 0.73 | 0.80 | 0.93 | 0.55 |
| Androgen Receptor (AR) | 0.60 | 0.98 | 0.97 | 0.98 | 0.96 | 0.97 | 0.98 | 0.96 | 0.97 | 0.98 | 0.98 | 0.98 | 0.98 | 0.97 | 0.98 | 0.98 | 0.98 |
| Androgen Receptor Ligand Binding Domain (AR-LBD) | 0.68 | 0.94 | 0.98 | 0.97 | 0.95 | 0.98 | 0.94 | 0.97 | 0.95 | 0.97 | 0.88 | 0.94 | 0.87 | 0.95 | 0.97 | 0.96 | 0.54 |
| Aromatase | 0.67 | 0.70 | 0.86 | 0.83 | 0.70 | 0.85 | 0.70 | 0.86 | 0.84 | 0.83 | 0.89 | 0.70 | 0.63 | 0.84 | 0.83 | 0.53 | 0.77 |
| Estrogen Receptor Alpha (ER) | 0.64 | 0.79 | 0.88 | 0.84 | 0.83 | 0.85 | 0.79 | 0.88 | 0.84 | 0.87 | 0.84 | 0.79 | 0.74 | 0.84 | 0.87 | 0.74 | 0.98 |
| Estrogen Receptor Ligand Binding Domain (ER-LBD) | 0.97 | 0.99 | 0.97 | 0.96 | 0.97 | 0.96 | 0.99 | 0.97 | 0.97 | 0.96 | 0.92 | 0.99 | 0.99 | 0.97 | 0.96 | 0.97 | 0.91 |
| Peroxisome Proliferator Activated Receptor Gamma (PPAR-Gamma) | 0.96 | 0.93 | 0.98 | 0.96 | 0.93 | 0.95 | 0.93 | 0.97 | 0.94 | 0.97 | 0.97 | 0.93 | 0.87 | 0.94 | 0.97 | 0.86 | 0.81 |
| Nuclear factor (erythroid-derived 2)-like 2/antioxidant responsive element (nrf2/ARE) | 0.91 | 0.82 | 0.84 | 0.82 | 0.76 | 0.83 | 0.82 | 0.85 | 0.84 | 0.84 | 0.80 | 0.82 | 0.85 | 0.84 | 0.84 | 0.89 | 0.81 |
| Heat shock factor response element (HSE) | 0.91 | 0.82 | 0.84 | 0.82 | 0.76 | 0.83 | 0.82 | 0.85 | 0.84 | 0.84 | 0.80 | 0.82 | 0.85 | 0.84 | 0.84 | 0.89 | 0.86 |
| Mitochondrial Membrane Potential (MMP) | 0.61 | 0.92 | 0.68 | 0.75 | 0.82 | 0.66 | 0.92 | 0.71 | 0.71 | 0.75 | 0.56 | 0.92 | 0.92 | 0.71 | 0.75 | 0.82 | 0.77 |
| Phosphoprotein (Tumor Supressor) p53 | 0.79 | 0.75 | 0.88 | 0.81 | 0.69 | 0.80 | 0.75 | 0.88 | 0.80 | 0.81 | 0.69 | 0.75 | 0.71 | 0.80 | 0.81 | 0.84 | 0.78 |
| ATPase family AAA domain-containing protein 5 (ATAD5) | 0.94 | 0.79 | 0.79 | 0.87 | 0.80 | 0.74 | 0.79 | 0.83 | 0.82 | 0.84 | 0.76 | 0.79 | 0.81 | 0.82 | 0.84 | 0.74 |  |

1. Banerjee P, Eckert AO, Schrey AK, Preissner R. ProTox-II: a webserver for the prediction of toxicity of chemicals. *Nucleic Acids Res*. 2018 Jul 2;46(W1):W257-W263.
